# TDP-43 induces mitochondrial damage and activates the mitochondrial unfolded protein response

**DOI:** 10.1371/journal.pgen.1007947

**Published:** 2019-05-17

**Authors:** Peng Wang, Jianwen Deng, Jie Dong, Jianghong Liu, Eileen H. Bigio, Marsel Mesulam, Tao Wang, Lei Sun, Li Wang, Alan Yueh-Luen Lee, Warren A. McGee, Xiaoping Chen, Kazuo Fushimi, Li Zhu, Jane Y. Wu

**Affiliations:** 1 State Key Laboratory of Brain and Cognitive Science, Institute of Biophysics, Chinese Academy of Sciences, Beijing, China; 2 University of Chinese Academy of Sciences, Beijing, China; 3 Department of Pathology & Neurology, The Cognitive Neurology & Alzheimer's Disease Center, Northwestern University Feinberg School of Medicine, Chicago, Illinois, United States of America; 4 National Institute of Biological Sciences, Beijing, China; 5 Center for Biological Imaging, Institute of Biophysics, Chinese Academy of Sciences, Beijing, China; 6 National Institute of Cancer Research, National Health Research Institutes, Miaoli, Taiwan; 7 Department of Neurology, Center for Genetic Medicine, Lurie Cancer Center, Northwestern University Feinberg School of Medicine, Chicago, Illinois, United States of America; University of California San Diego, UNITED STATES

## Abstract

Mutations in or dys-regulation of the TDP-43 gene have been associated with TDP-43 proteinopathy, a spectrum of neurodegenerative diseases including Frontotemporal Lobar Degeneration (FTLD) and Amyotrophic Lateral Sclerosis (ALS). The underlying molecular and cellular defects, however, remain unclear. Here, we report a systematic study combining analyses of patient brain samples with cellular and animal models for TDP-43 proteinopathy. Electron microscopy (EM) analyses of patient samples revealed prominent mitochondrial impairment, including abnormal cristae and a loss of cristae; these ultrastructural changes were consistently observed in both cellular and animal models of TDP-43 proteinopathy. In these models, increased TDP-43 expression induced mitochondrial dysfunction, including decreased mitochondrial membrane potential and elevated production of reactive oxygen species (ROS). TDP-43 expression suppressed mitochondrial complex I activity and reduced mitochondrial ATP synthesis. Importantly, TDP-43 activated the mitochondrial unfolded protein response (UPR^mt^) in both cellular and animal models. Down-regulating mitochondrial protease LonP1 increased mitochondrial TDP-43 levels and exacerbated TDP-43-induced mitochondrial damage as well as neurodegeneration. Together, our results demonstrate that TDP-43 induced mitochondrial impairment is a critical aspect in TDP-43 proteinopathy. Our work has not only uncovered a previously unknown role of LonP1 in regulating mitochondrial TDP-43 levels, but also advanced our understanding of the pathogenic mechanisms for TDP-43 proteinopathy. Our study suggests that blocking or reversing mitochondrial damage may provide a potential therapeutic approach to these devastating diseases.

## Author summary

TDP-43 proteinopathy is a group of fatal neurological diseases. Here, we report a systematic examination of the role of mitochondrial damage in TDP-43 proteinopathy using patient brain tissues, as well as cellular and animal models. Our data show that TDP-43 induces severe mitochondrial damage, accompanied by activation of UPR^mt^ in both cellular and animal models of TDP-43 proteinopathy. LonP1, one of the key mitochondrial proteases in UPR^mt^, protects against TDP-43 induced cytotoxicity and neurodegeneration. Our study uncovers LonP1 as a modifier gene for TDP-43 proteinopathy and suggests protecting against or reversing mitochondrial damage as a potential therapeutic approach to these neurodegenerative disorders.

## Introduction

TDP-43 proteinopathy is characterized by the presence of TDP-43 immunoreactive inclusion bodies in the affected tissues. Clinically, TDP-43 proteinopathy manifests as a spectrum of different neurodegenerative diseases, ranging from dementia (especially fronto-temporal lobar degeneration, FTLD) and motor neuron disease (MND) to traumatic brain injuries [1–4]. FTLD is a prevalent form of dementia with progressive atrophy of the frontal and/or temporal cortices [5–7]. Amyotrophic Lateral Sclerosis (ALS), a common form of MND, is characterized by a progressive loss of upper and lower motor neurons [8–10]. TDP-43 associated neurodegenerative diseases are clinically and genetically heterogeneous. A significant fraction of ALS patients exhibit cognitive impairment [11,12]; and ~15% of FTLD patients also show locomotor defects and meet the diagnostic criteria for ALS [12,13]. TDP-43-positive lesions are the most frequently identified pathology among FTLD and ALS cases and also present in ~50% AD samples [14–16]. However, the pathogenic mechanisms underlying TDP-43 proteinopathy remain unclear.

Mitochondrial damage is associated with a range of neurodegenerative diseases, including Alzheimer’s disease (AD), Parkinson’s disease (PD) and MNDs [17–19]. Mitochondrial changes have been detected in cellular and animal models for TDP-43 proteinopathy [16,20–27]. It was recently reported that suppressing mitochondrial localization of TDP-43 blocked TDP-43 neurotoxicity [28]. However, mitochondrial morphological changes have not yet been characterized in patient samples, and the effects of TDP-43 on mitochondrial function remain controversial [27–29].

To maintain mitochondrial homeostasis, cells sense and respond to mitochondrial damage by activating a program known as the mitochondrial unfolded protein response (UPR^mt^), which includes induction of mitochondrial chaperones assisting in proper protein folding, and of proteases promoting clearance of misfolded proteins [30–32]. Recent studies suggest a role of UPR^mt^ in Alzheimer’s disease, Parkinson’s disease and ALS-SOD [33–35]. However, the role of UPR^mt^ in TDP-43 proteinopathy has not been reported.

Here, we present a systematic study of TDP-43 proteinopathy combining cellular and animal models with patient samples. Analyses using electron microscopy (EM) reveal prominent mitochondrial damage in brain tissues from TDP-43 proteinopathy patients. These mitochondrial impairments include swollen and degenerated cristae or a complete loss of cristae. Similar mitochondrial cristae changes are detected in our cellular and animal models. Consistently, mitochondrial functional impairments are observed, including decreased mitochondrial membrane potential, reduced mitochondrial ATP synthesis and elevated mitochondrial ROS production. Our data show that mitochondrial impairment induced by TDP-43 is an early event, preceding cell death. Furthermore, induced TDP-43 expression leads to the activation of UPR^mt^ in both cellular and fly models for TDP-43 proteinopathy. LonP1, one of the key mitochondrial proteases in UPR^mt^, plays an important role in the degradation of mitochondrial TDP-43. Consistent with the mRNA changes of LonP1 in cellular and fly models, LonP1 protein levels are increased in a fraction of the brain samples of patients affected by FTLD-TDP. Importantly, down-regulation of LonP1 in TDP-43 expressing flies not only induces more severe mitochondrial damage, but also advances disease onset and exacerbates the neurodegeneration phenotype in the animal model. These results suggest that LonP1 plays a protective role against TDP-43-induced neurotoxicity, especially at an early stage of the disease. Together, our data demonstrate that mitochondrial damage is a critical feature of TDP-43 proteinopathy and suggest that protecting mitochondria may have therapeutic potential.

## Results

### Mitochondrial impairment in the brain samples of TDP-43 proteinopathy patients

To investigate the role of mitochondria in TDP-43 proteinopathy, we examined mitochondrial morphology in brain samples from patients using transmission electron microscopy (TEM) and immuno-electron microscopy (IEM). Following resin-embedding to obtain clear images of mitochondria, we analyzed brain samples from five patients with the pathological diagnosis of either FTLD-TDP or ALS-FTLD-TDP, together with the samples from three control subjects without any TDP-43 pathology (for details, see S1 Table).

**Fig 1:**
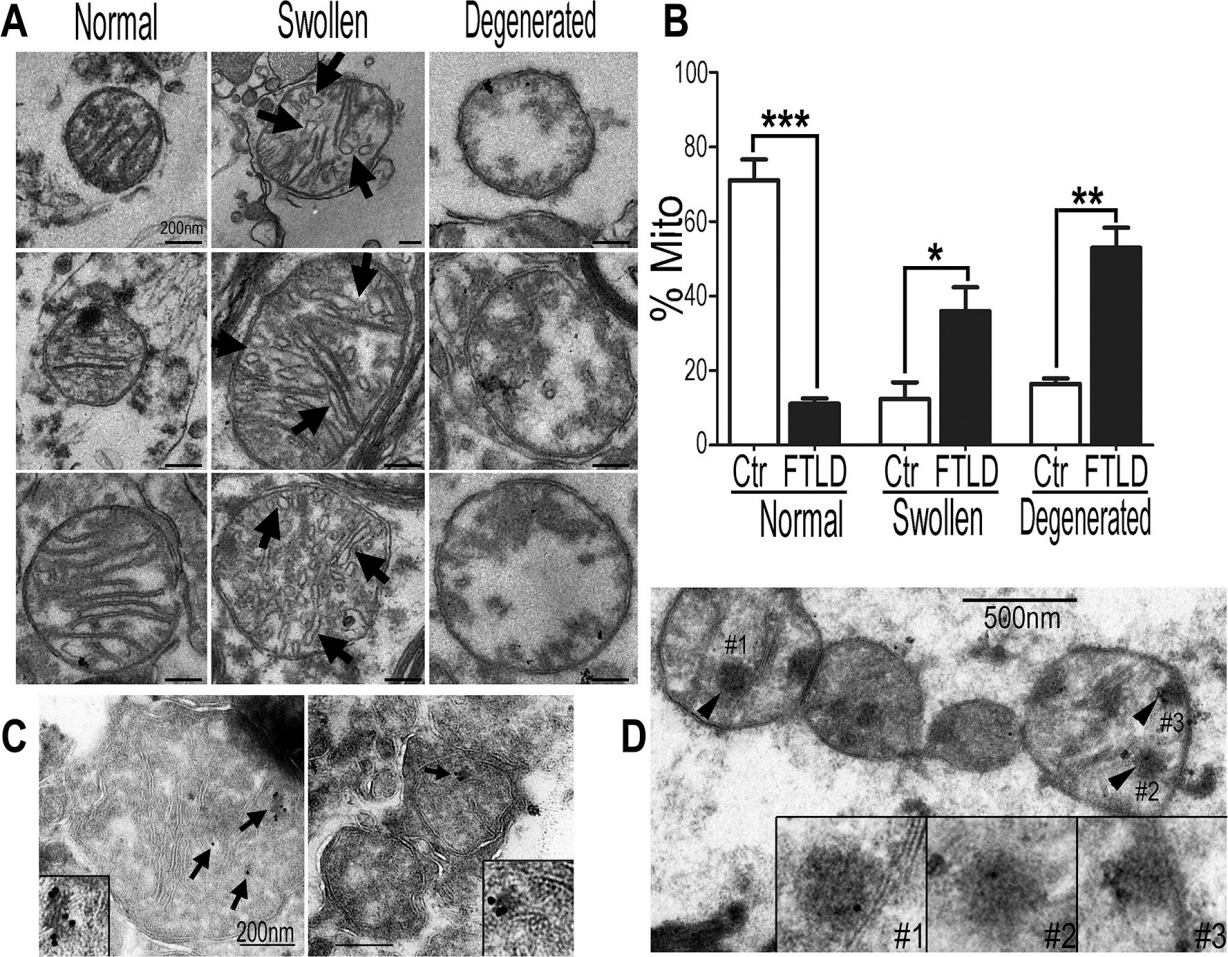
Mitochondrial changes in brain samples of TDP-43 proteinopathy patients. (**A**) EM images showing mitochondrial changes. The majority of mitochondria in the control brain samples have organized tubular cristae (left panels), whereas damaged mitochondria showing vesicular or swollen cristae (middle panels; arrows) or a marked loss of cristae (“degenerated” type; in the right panels) were frequently detected in the brain tissues of FTLD-TDP patients. Scale bars: 200nm (**B**) Quantification of the mitochondrial damage in the control and FTLD-TDP brain samples (all samples were from temporal cortices, except one from hippocampus and one from dentate gyrus; for details, see S1 Table). More than 100 mitochondria were analyzed from each sample, including 3 controls (Ctr: 123, 147, 118 mitochondria) and 5 FTLD-TDP brain samples (FTLD: 112, 236, 115, 240, 184 mitochondria). (**C**) Immuno-electron microscopy (IEM) using the specific anti-TDP-43 antibody of brain tissues, from either a control subject (left panel) or an FTLD-TDP patient (right panel), revealed endogenous TDP-43 inside mitochondria. (**D**) Electron-dense TDP-43 positive protein aggregates were detected inside ~1% of mitochondria from FTLD-TDP patients' samples, but not in any control samples. Data were analyzed using a Student’s *t*-test (*: *P*<0.05; **: *P*<0.01; ***: *P*<0.001).

The majority of mitochondria in the control brain tissues showed normal morphology, with intact mitochondrial membrane and well-organized cristae (left panels in Fig 1A). In contrast, more than 80% of mitochondria in the patient brains exhibited significant mitochondrial damage, especially abnormal cristae structure (Fig 1). Abnormal mitochondrial cristae presented as either a “vesicular” type with swollen cristae (marked by the arrows in the middle panels of Fig 1A; as “Swollen” in Fig 1A and Fig 1B) or a “degenerated” type with a partial to complete loss of cristae (the right panels of Fig 1A; as “Degenerated” in Fig 1A and Fig 1B). Damaged mitochondria were significantly increased in all 5 FTLD-TDP brains as compared with the control brains (Fig 1B).

IEM analyses of the brain tissues using a specific anti-TDP-43 antibody revealed that TDP-43 immunostaining signals were clearly detected inside mitochondria in the brain samples of both control and FTLD-TDP patients (marked by arrows in Fig 1C; with enlarged views in insets), demonstrating that the endogenous TDP-43 protein is localized inside mitochondria, consistent with a recent report [28]. Interestingly, electron-dense TDP-43 positive protein aggregates were detected inside ~1% of mitochondria in FTLD-TDP patient samples (arrowheads in Fig 1D), but were not detected in any control samples. These EM analyses demonstrate that mitochondrial damage is a prominent feature in the pathology of brain tissues of TDP-43 proteinopathy patients.

### Mitochondrial impairment in a cellular model of TDP-43 proteinopathy

**Fig 2:**
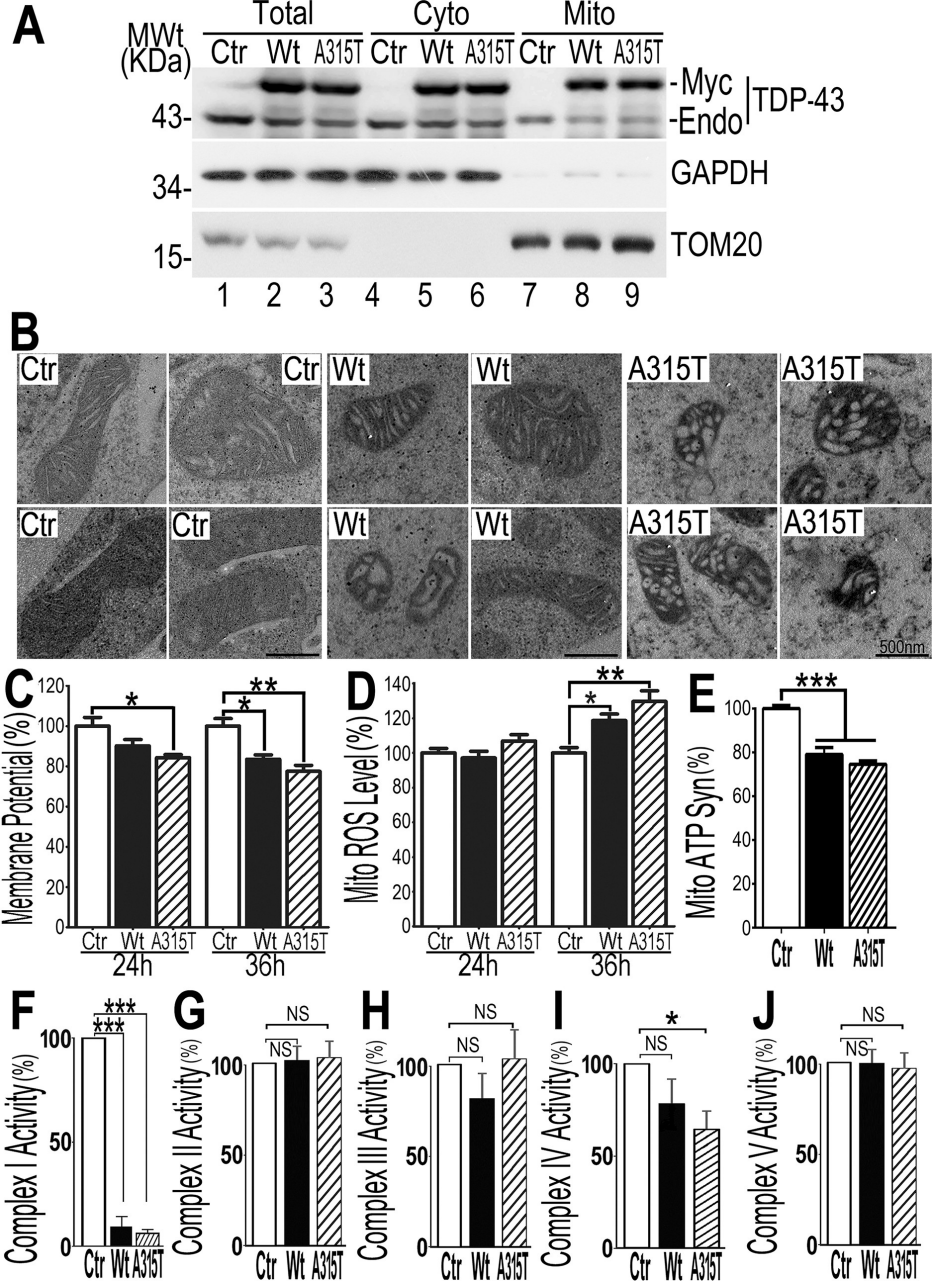
TDP-43 is localized to mitochondria, induces mitochondrial damage and suppresses mitochondrial complex I and reduces mitochondrial ATP synthesis. (**A**) TDP-43 is detected in purified mitochondria. Highly purified mitochondria were prepared from HEK293 cells expressing either the control vector, or Wt, or A315T-mutant TDP-43 protein following tetracycline (Tet; 24 hr) induction. The purity of the mitochondrial preparation was confirmed by the detection of mitochondrial TOM20 and the absence of the cytoplasmic GAPDH protein. (**B**) TEM micrographs showed mitochondrial abnormalities (decreased size and abnormal cristae) in HEK293 cells expressing the Wt or A315T-mutant TDP-43 protein 24 hr post-induction, as compared with control cells (Ctr). It should be noted that there was no detectable cell death at this time point. Scale bars: 200 nm. (**C, D**) Quantification of mitochondrial membrane potential and ROS levels in cells expressing Wt- or A315T-mutant TDP-43 as compared with control cells, 24 or 36 hr post-induction. Cells were stained using JC1 (C) or mitoSOX-red (D) respectively and analyzed using flow cytometry. (**E**) Mitochondrial ATP synthesis was decreased in cells expressing either Wt or A315T-mutant TDP-43 protein. Mitochondrial ATP synthesis was measured in the mitochondria purified from cells expressing Wt or A315T-mutant TDP-43 as compared with control cells (Ctr) 36 hr post-induction. (**F-J**) Changes in activities of mitochondrial complexes I-IV 24 hr post-induction. (**F**) Expression of either Wt or A315T-mutant TDP-43 significantly reduced mitochondrial complex I activity. (**G, H**) Expression of either Wt or A315T-mutant TDP-43 did not affect mitochondrial complex II or III activity. (**I)** Expression of A315T-mutant TDP-43 reduced mitochondrial complex IV activity. **(J**) Expression of either Wt or A315T-mutant TDP-43 did not affect mitochondrial complex V activity. Data in all panels represent 3 independent experiments [one-way ANOVA with Bonferroni post hoc test (ns: *P*>0.05; *: *P*<0.05; **: *P*<0.01; ***: *P*<0.001)].

To investigate the effects of TDP-43 on mitochondrial morphology and function in living cells, we established tetracycline (Tet) inducible HEK293 cell lines, expressing either wild type (Wt) or an ALS-associated TDP-43 mutant (A315T). Following Tet-induction for 24 hr, total cell lysates, cytoplasmic fractions and purified mitochondrial preparations were examined by Western blotting. The purity of the mitochondrial preparation was confirmed by the detection of mitochondrial protein TOM20 and the absence of the cytoplasmic GAPDH protein. Consistent with the IEM data from the human brain samples, the endogenous TDP-43 as well as the exogenously expressed Wt or ALS-mutant (A315T) TDP-43 were detected in purified mitochondria (Fig 2A; for a longer exposure, see S1A Fig), supporting the mitochondrial localization of the TDP-43 protein. Consistent with previous studies [36,37], expression of the exogenous TDP-43 suppressed expression of the endogenous TDP-43 (marked by “Endo” in Fig 2A).

We next performed EM analyses of HEK293 cells expressing TDP-43 to characterize mitochondrial changes. In control cells, the vast majority of mitochondria exhibited normal morphology, with well-organized cristae (Fig 2B). However, in cells expressing the A315T-mutant TDP-43, severe mitochondrial damage was detected, with significantly reduced mitochondrial sizes and impaired mitochondrial cristae 24 hr post-induction. When Wt TDP-43 was expressed, similar mitochondrial damage was also detected, although to a lesser extent (Fig 2B; see S1B and S1C Fig). These data indicate that expression of Wt or ALS-mutant TDP-43 protein leads to mitochondrial damage in cultured cells.

### TDP-43 induced mitochondrial dysfunction precedes cell death

To examine the temporal relationship between TDP-43-induced mitochondrial damage and cell death, we carried out a series of experiments using the Tet-inducible cells expressing Wt- or A315T-mutant TDP-43 proteins at different time points (0, 24 or 36 hr) following induction of TDP-43 expression. We first measured mitochondrial membrane potential, ROS production and ATP synthesis (Fig 2C–2E). Cells were stained with JC1 (a mitochondrial membrane potential indicator), or mitoSOX red fluorescent dye (a mitochondrial ROS indicator), and then analyzed by flow cytometry. Mitochondrial membrane potential began to show a reduction at 24 hr post-induction in cells expressing A315T-mutant TDP-43; and by 36 hr post-induction, mitochondrial membrane potential reduction was detected in cells expressing either Wt or A315T-mutant TDP-43 (Fig 2C). By 36 hr following the induction of expression of Wt or A315T-mutant TDP-43, the mitochondrial ROS level was significantly increased (Fig 2D). Total cellular ATP levels and mitochondrial ATP synthesis were measured following published protocols [38,39]. Thirty-six hr following induction of TDP-43 expression (Fig 2E), total cellular ATP level did not change (S2A Fig). However, mitochondrial ATP synthesis at this time point was significantly reduced in cells expressing either Wt or A315T-mutant TDP-43 (with ~20% and ~25% decrease in the Wt and A315T-groups respectively), as compared with the control group (Fig 2E).

To understand the mechanism by which increased TDP-43 expression suppressed mitochondrial ATP synthesis, we examined which mitochondrial complexes (complex I through V) in oxidative phosphorylation were affected. Interestingly, complex I activity was significantly reduced by 24 hr following induction of either Wt or A315T mutant TDP-43 (Fig 2F); complex IV activity was also reduced by the expression of A315T-mutant TDP-43 (Fig 2I). In contrast, the activities of complexes II, III (Fig 2G, Fig 2H) and complex V (Fig 2J) were unaffected. These data indicate that increased TDP-43 expression impairs mitochondrial ATP synthesis, possibly by suppression of mitochondrial complex I. TDP-43-induced reduction in the complex I activity was not likely the result of overall suppression of complex I genes by TDP-43, because quantitative PCR analyses of a number of complex I genes did not show a general reduction in the expression of these genes (see S2B Fig). Future experiments are necessary to elucidate the mechanism by which TDP-43 suppresses the activity of complex I.

**Fig 3:**
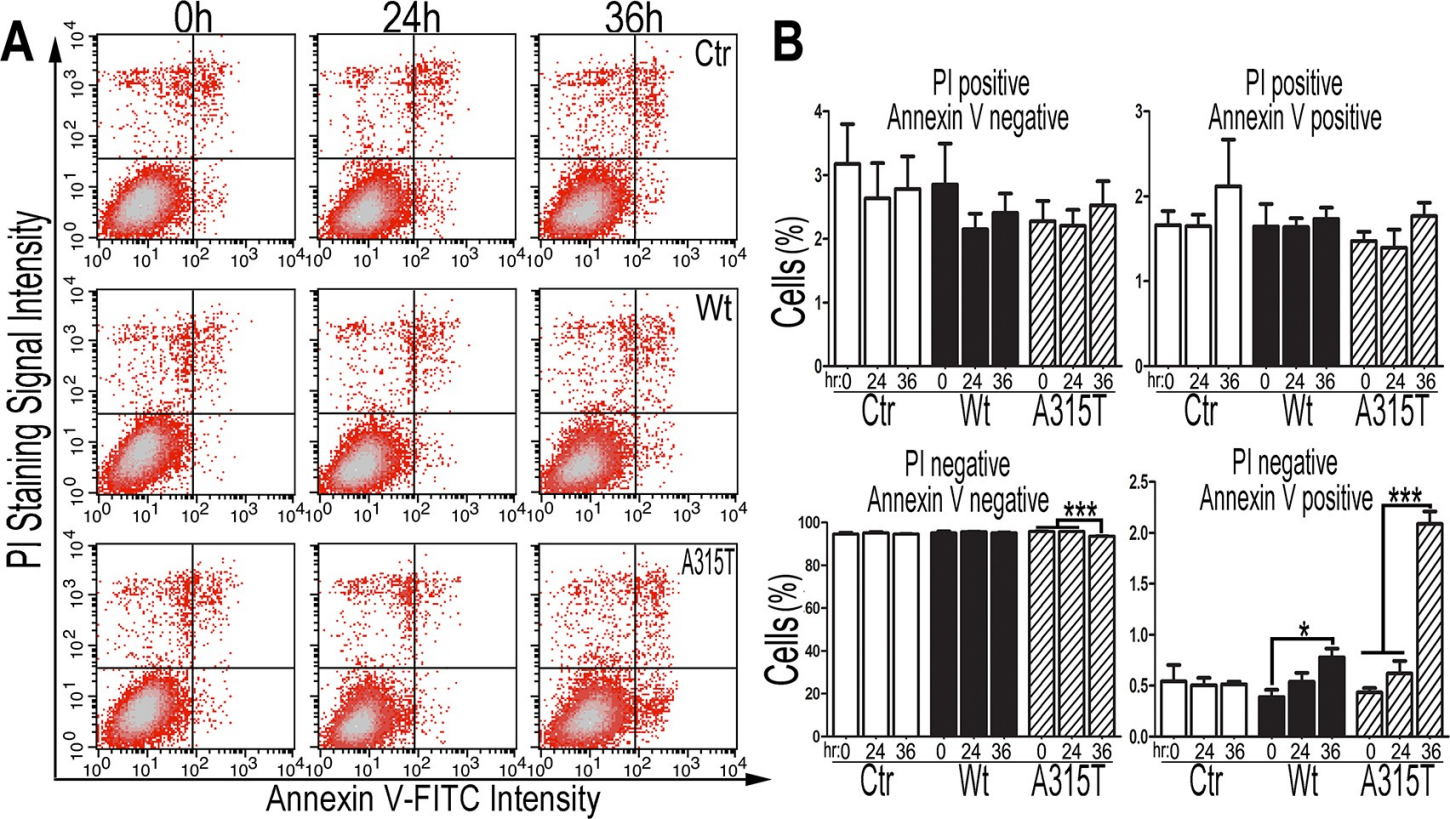
Expression of the Wt or A315T-mutant TDP-43 induces cell death in inducible stable cells. (**A**) FACS analyses of cells following staining using an Annexin V-FITC/PI kit to quantify cell death in HEK293 cells following induction of TDP-43 expression. (**B**) Quantification of the percentage of different cell populations at different time points. At 36 hr post-Tet induction, the expression of either Wt or A315T-mutant TDP-43 protein induced apoptosis (Annexin V positive and PI negative) but not necroptosis (Annexin V negative and PI positive) or late apoptosis (Annexin V positive and PI positive). The data were analyzed using a one-way ANOVA with Bonferroni post hoc test (representing 4 independent experiments; *: *P*<0.05; **: *P*<0.01; ***: *P*<0.001).

To examine cell death, cells were stained with an Annexin V-FITC/PI (propidium idodide) kit followed by flow cytometry analyses (Fig 3). Annexin V-positive/PI-negative, Annexin V-negative/PI-positive or Annexin V-positive/PI-positive staining indicates apoptosis, necroptosis or late apoptosis/necroptosis, respectively. Up to 36 hr post-induction, Annexin V-negative/PI-positive or PI/Annexin V double-postive cell populations did not show significant changes in TDP-43 expressing cells compared to the control group. Cells expressing A315T-mutant TDP-43 showed significantly increased cell death only after 36 hr post-induction of TDP-43 expression (~2% cells showing Annexin V-positive/PI-negative staining; compared with ~0.5% in the control cells); whereas cells expressing wild type TDP-43 showed a less dramatic increase in cell death, also only after 36 hr post-induction (Fig 3A, Fig 3B). It should be noted that at this time point only a small fraction (<5%; estimated by biochemical fractionation) of the total TDP-43 was detected in purified mitochondria (possibly due to the efficient degradation of mitochondrial TDP-43 before the disruption of the balanced mitochondrial proteostasis). Because mitochondrial dysfunction was observed at the 24 hr time point, these results demonstrate that TDP-43-induced mitochondrial dysfunction is an early event preceding cell death, suggesting that mitochondrial impairment may contribute to TDP-43 cytotoxicity.

### TDP-43 induces mitochondrial damage and increases mitochondrial ROS production in a transgenic fly model of TDP-43 proteinopathy

**Fig 4:**
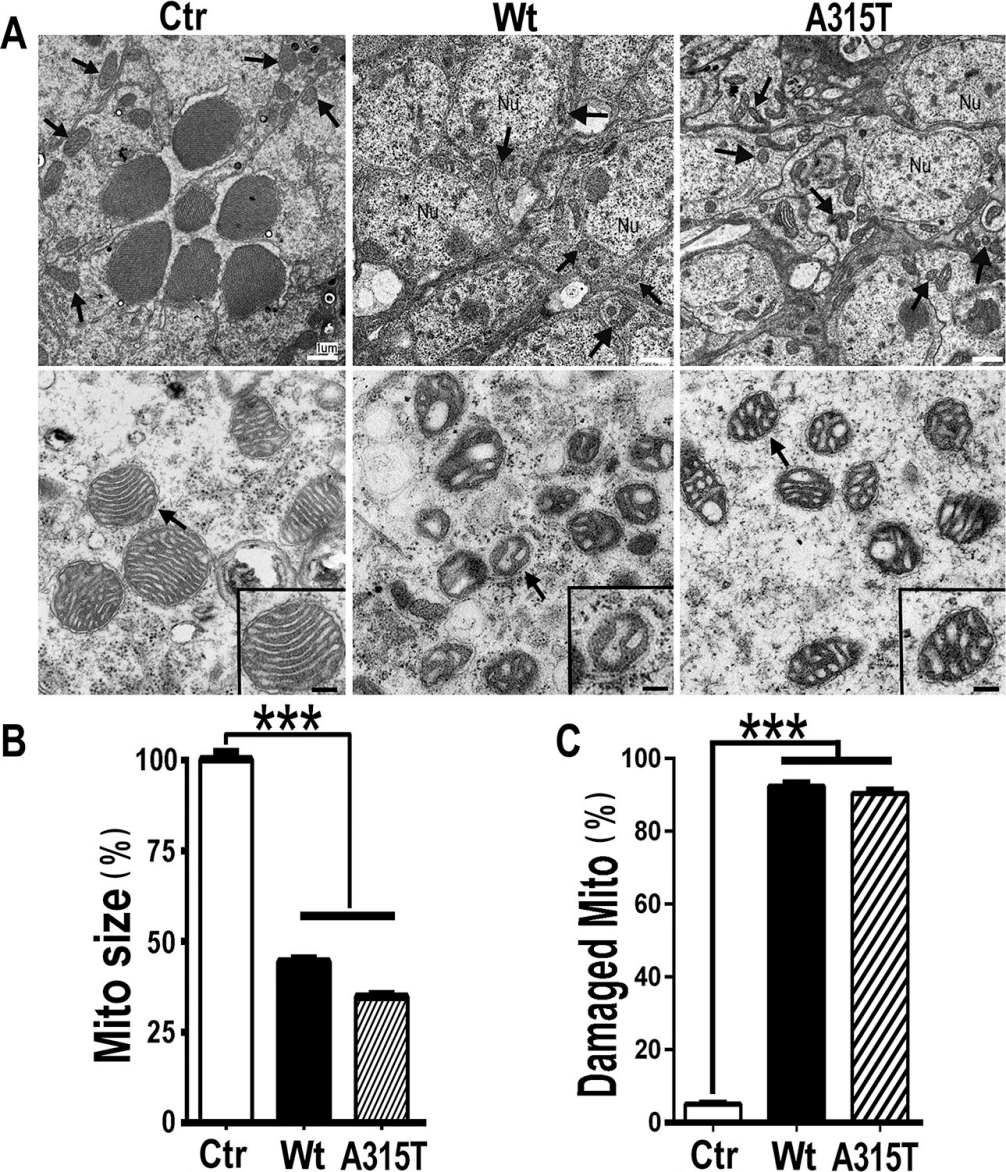
Mitochondrial defects in transgenic flies expressing TDP-43. (**A**) TEM micrographs show intact ommatidial structures with 7-rhabdomere trapezoids in control fly eyes (3-day-old female adult fly), whereas the rhabdomere structures are severely damaged or completely lost in the retinae of flies expressing either Wt or A315T-mutant TDP-43 protein. Sections 10–40 μm beneath the cornea were used to show cross-sections of the photoreceptors as described previously [88]. Mitochondria are marked by arrows. Scale bars: 1um. In the lower panels are higher-magnification TEM images, showing that mitochondria in fly retinae expressing either Wt, or A315T-mutant TDP-43 are smaller in size and have abnormal cristae as compared with those in the control flies. Higher-magnification images of representative mitochondria (arrows) were shown in the insets of the corresponding panels. Scale bars: 200nm. (**B**) Quantification of mitochondrial sizes in the corresponding groups. Data from 3 independent experiments were analyzed using a one-way ANOVA with Bonferroni post hoc test (***: *P*<0.001). (**C**) Quantification of damaged mitochondria in different groups with percentage of mitochondria showing abnormal cristae. Two independent fly retinae were examined by TEM in each group: control flies (Ctr), or flies expressing either Wt or A315T-mutant TDP-43 in their photoreceptors. More than 200 mitochondria in each group (227 in Ctr, 274 in Wt, and 270 in A315T groups) were examined. Data from 3 independent experiments were analyzed using a one-way ANOVA with Bonferroni post hoc test (***: *P*<0.001). Fly genotypes: **Ctr**: GMR-Gal4/UAS-RFP; **Wt**: GMR-Gal4/UAS-Wt-TDP-43-RFP; **A315T**: GMR-Gal4/UAS-A315T-TDP-43-RFP.

To investigate TDP-43-induced mitochondrial damage *in vivo*, we examined transgenic flies expressing either Wt or A315T-mutant TDP-43 reported in our previous studies [40–42]. Transmission EM analyses of control fly eyes in 3-day old adult animals revealed intact ommatidial structures with seven rhabdomeres, whereas expression of either Wt or A315T-mutant TDP-43 in fly eyes led to severe ommatidial defects, often with a complete loss of rhabdomeres (Fig 4A). Mitochondria in fly eyes expressing either Wt or A315T-mutant TDP-43 showed a significant decrease in size when compared with control flies (Fig 4). Importantly, more than 85% of mitochondria in the photoreceptors expressing Wt or ALS-mutant TDP-43 exhibited swollen or vesicular cristae, whereas only ~5% of mitochondria in the control group showed damage (Fig 4A and Fig 4C). In this setting, TDP-43 was expressed in photoreceptors under a strong GMR-Gal4 driver from an early stage, leading to rapid and severe mitochondrial damage. By the time of EM examination, >85% mitochondria showed damage in both Wt and A315T-mutant groups, not allowing us to detect differences between the two groups. It is remarkable that mitochondria in fly photoreceptors expressing either Wt or A315T-mutant TDP-43 showed similar mitochondrial cristae damage as those detected in the brain tissues of TDP-43 proteinopathy patients (see Fig 1A). To examine whether the results observed were due to developmental defect(s), we used a system in which TDP-43 expression was induced only in adulthood using a temperature-sensitive tubulin-Gal80ts promoter with the GMR-Gal4 photoreceptor-specific driver or the Elav-Gal4 pan-neuronal driver (see S3 Fig). In this system, flies expressing A315T-mutant TDP-43 in photoreceptors following heat shock induction at the adult stage indeed exhibited progressive mitochondrial damage and retinal degeneration (S3 Fig).

The mitochondrion is a major source for the production of reactive oxygen species (ROS) [43]. Mitochondrial dysfunction can lead to the accumulation of ROS [44]. Furthermore, excessive ROS production affects neuronal survival and function [45,46]. We therefore examined whether TDP-43 expression affected mitochondrial ROS production *in vivo* using transgenic flies expressing TDP-43 in motor neurons. A fly line expressing mito-roGFP-Grx1, an *in vivo* mitochondrial ROS reporter [47], was crossed with either control RFP or TDP-43-RFP expressing flies. Ratiometric fluorescence confocal imaging was carried out to measure mitochondrial ROS levels in motor neurons expressing control (RFP) or TDP-43-RFP using a previously published protocol [47]. Significantly elevated mitochondrial ROS levels were detected in motor neurons expressing either Wt- or A315T-mutant TDP-43 as compared with the control group (see S4 Fig), indicating that TDP-43 expression in motor neurons resulted in mitochondrial dysfunction.

### Mitochondrial unfolded protein response is activated in cellular and animal models of TDP-43 proteinopathy

Our results presented above showed that increased TDP-43 expression led to mitochondrial cristae damage, reduced activities of mitochondrial OXPHOS complex I and IV, as well as decreased mitochondrial ATP synthesis. In addition, TDP-43 immuno-reactive aggregates were detected inside mitochondria of FTLD-TDP patient brain samples. These observations prompted us to examine if TDP-43 activated the mitochondrial unfolded protein response (UPR^mt^).

**Fig 5:**
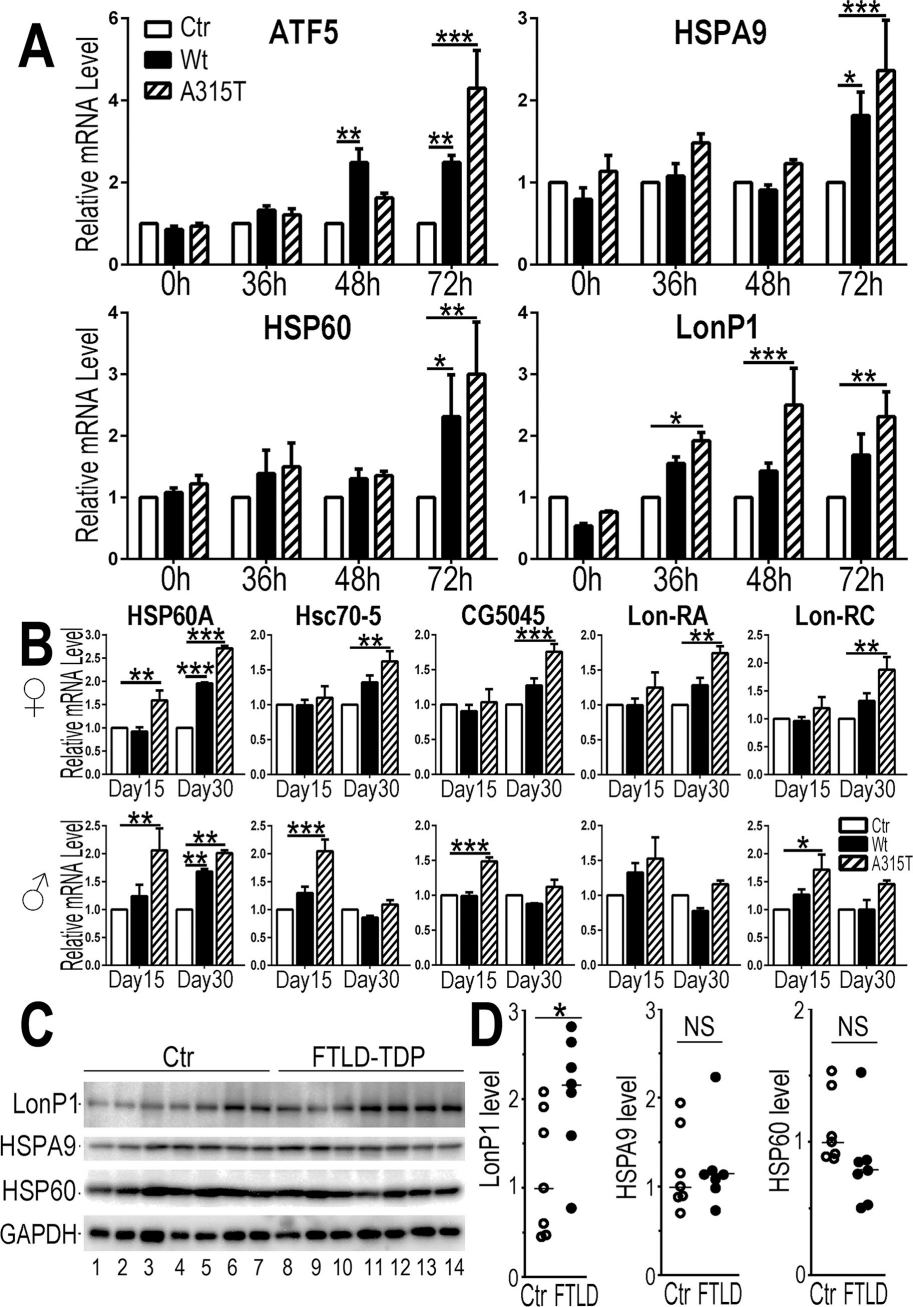
Increased expression of UPR^mt^ genes in HEK293 cells and flies expressing TDP-43, and elevated LonP1 protein in FTLD-TDP brains. (**A**) Expression levels of UPR^mt^ related genes, including ATF5, HSPA9 (mtHSP70), HSP60 and LonP1, as detected by qPCR in TDP-43 inducible stable cells at different time points following induction of TDP-43 expression: 0, 36, 48 and 72-hr time points. HPRT1 was used as an internal control. (**B**) Expression levels of UPR^mt^ related *Drosophila* genes in female and male flies, including HSP60, Hsc70-5 (mtHSP70), CG5045 (ClpP homolog) and Lon (Lon-RA and Lon-RC isoforms), as detected by qPCR in Elav-Gal4/Tub-Gal80^ts^ driven TDP-43 transgenic flies at day 15 and day 30 post-induction of TDP-43 expression. Actin 5C was used as an internal control. Fly genotypes: **Ctr**: Elav-Gal4/Tub-Gal80^ts^ /UAS-RFP; **Wt**: Elav-Gal4/Tub-Gal80^ts^/UAS-Wt-TDP-43-RFP; **A315T**: Elav-Gal4/Tub-Gal80^ts^ /UAS-A315T-TDP-43-RFP. Data from 3 independent experiments (panels A and B) were analyzed using a two-way ANOVA with Bonferroni post hoc test (*:*P*<0.05; **:*P*<0.01; ***: *P*<0.001). (**C-D**) Western blotting analyses using brains from control or FTLD-TDP patients show that LonP1 protein levels were higher in brain samples from patients affected by FTLD-TDP as compared with the control subjects. The levels of HSPA9 and HSP60 proteins were not changed in the patient brains. These brain samples have been reported previously [40]. Data were analyzed using StatPlus with a Student’s *t*-test (*:*P*<0.05; NS: not significant).

Using our inducible HEK293 cells expressing Wt or A315T-mutant TDP-43, we examined mRNA levels of known genes critical for UPR^mt^, including ATF5, HSPA9 (mtHSP70), HSP60 and LonP1. Quantitative RT-PCR analyses revealed that by 48 hr post-induction of TDP-43 expression, mRNA levels of ATF5 and LonP1 were increased, and that by 72 hr post-induction, mRNA levels of ATF5, HSPA9, HSP60 and LonP1 were all increased in cells expressing either Wt- or A315T-mutant TDP-43 (Fig 5A). To investigate whether TDP-43 expression activated UPR^mt^ *in vivo*, we induced TDP-43 expression in transgenic flies at the adult stage by heat shock using Elav-Gal4 pan-neuronal driver containing a temperature-sensitive tubulin-Gal80ts element, Elav-Gal4/tubulin-Gal80^ts^ driver [48] (see S3A Fig). At day 15 and day 30 after induction of TDP-43 expression, fly heads were collected for qRT-PCR analyses (Fig 5B). In female flies, by day 15 post-induction, HSP60A mRNA level was significantly increased in A315T-mutant expressing flies; and by day 30 post-induction, mRNA levels of HSP60A, Hsc-70-5, CG5045 (encoding ClpP) and two isoforms of Lon (the Drosophila ortholog of mammalian LonP1) were increased in TDP-43 expressing flies, especially those expressing A315T-mutant TDP-43. In male flies, the mRNA levels of all four genes were increased in flies expressing A315T-mutant TDP-43, and to a lesser extent in flies expressing Wt TDP-43, at 15 day post-induction. However, increased expression of only HSP60A, but not other three genes, was detected by day 30 post-induction of TDP-43 expression (Fig 5B). These data support that UPR^mt^ is activated by TDP-43 expression in the fly model for TDP-43 proteinopathy. Future studies are necessary to understand the significance of and mechanisms underlying the gender different responses observed in TDP-43 flies.

We next examined if protein levels of these UPR^mt^ genes are altered in TDP-43 proteinopathy patient samples using a panel of brain samples characterized previously [40]. Western blotting analyses indicate that the average level of LonP1 protein in TDP-43 proteinopathy patient brains was higher than that in the control brains (Fig 5C, Fig 5D). This is consistent with the possibility that UPR^mt^ may be activated in a subset of TDP-43 proteinopathy patient brains. There was no significant difference between patient and control samples in the protein levels of either HSPA9 or HSP60. Together, these results support the notion that UPR^mt^ is activated in cellular and animal models of TDP-43 proteinopathy as well as a subset of FTLD-TDP patient brains.

### LonP1 interacts with TDP-43 and reduces the mitochondrial TDP-43 protein level

**Fig 6:**
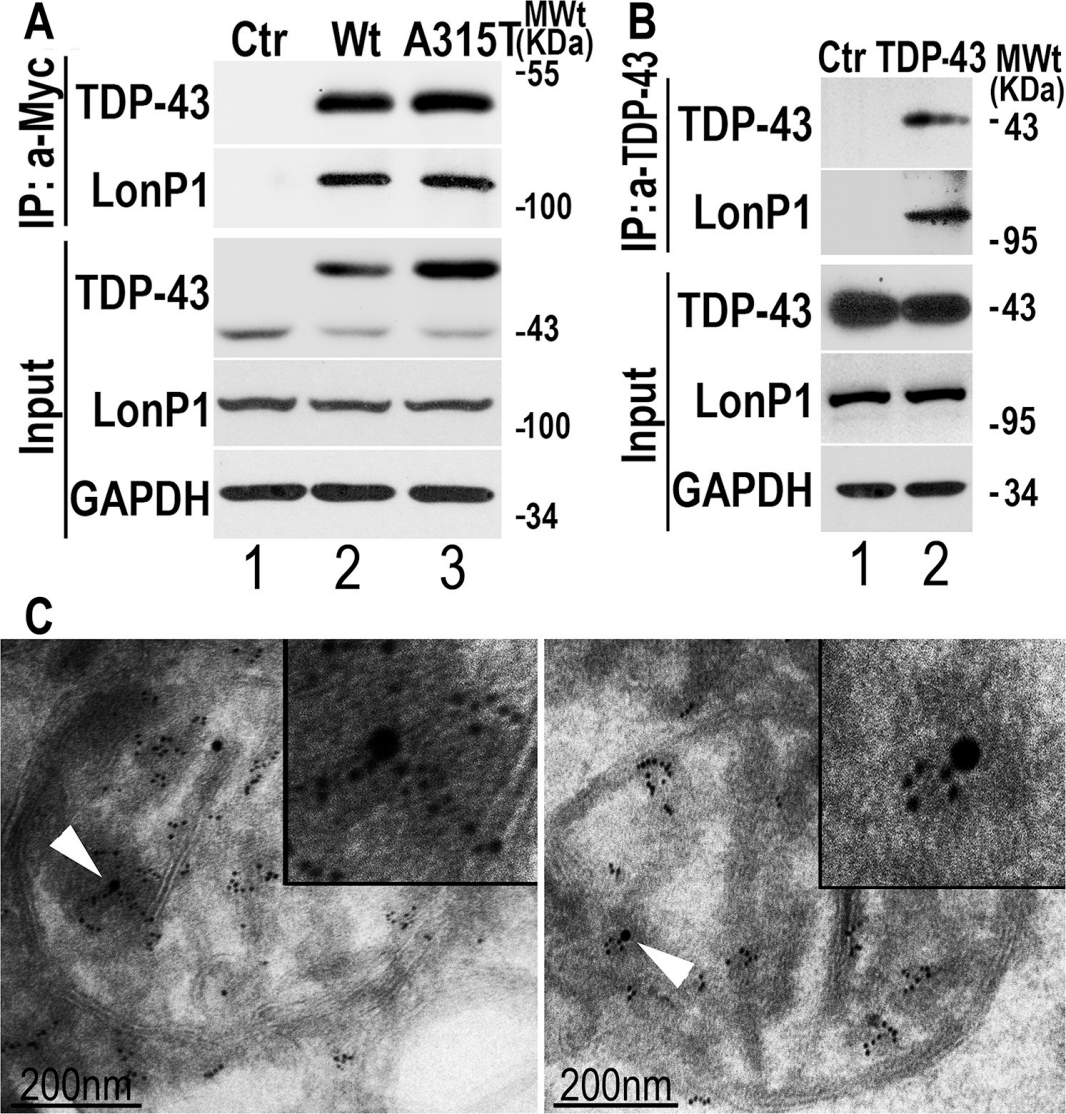
TDP-43 interacts with LonP1. **(A, B) Interaction of TDP-43 with LonP1 as detected by co-immunoprecipitation (coIP) assay. (A)** Immunoprecipitation experiment was performed using a monoclonal anti-Myc antibody with cell lysates from HEK293 stable inducible cells expressing vector control (Ctr) or Myc-tagged Wt or A315T-mutant TDP-43 (36 hr following Tet-induction). Western blotting experiments were carried out using specific anti-LonP1 or TDP-43 or Myc antibodies as indicated in cell lysates or immunoprecipitated proteins. (**B) The endogenous TDP-43 protein interacts with LonP1.** Immunoprecipitation experiment was performed with HEK293 cell lysates using the specific anti-TDP-43 antibody or non-specific IgG as a control (Ctr). LonP1 was detected by WB in immunoprecipitated proteins by anti-TDP-43, but not in that by the IgG control. Data in panels A and B represent three independent experiments. **(C) IEM analyses reveal co-localization of TDP-43 and LonP1 immunostaining signals inside mitochondria**. IEM was carried out to examine mitochondria in brain tissue of FTLD-TDP patients using murine anti-TDP-43 and rabbit anti-LonP1 followed by anti-murine- 6nm gold particles and anti-rabbit-15nm gold particles.

We further examined the relationship between LonP1 and TDP-43. LonP1 is a major mitochondrial matrix protease and a member of the evolutionarily conserved superfamily of AAA+ ATPases. LonP1 plays a critical role in mitochondrial protein quality control by preferentially degrading misfolded or oxidized proteins [49]. We first tested whether TDP-43 interacted with LonP1 in a co-immunoprecipitation assay using an anti-Myc antibody in cells expressing Myc-tagged TDP-43. LonP1 was detected among immunoprecipitated proteins from cell lysates expressing either Wt or A315T-mutant TDP-43, but not the control lysates (Fig 6A), suggesting that LonP1 interacted with TDP-43. Further co-immunoprecipitation experiments using a specific TDP-43 antibody showed that the endogenous TDP-43 and LonP1 proteins interacted with each other (Fig 6B). To examine if TDP-43 protein co-localized with LonP1 inside mitochondria, we performed immuno-electron microscopy (IEM) using FTLD-TDP brain samples. In these brain samples, TDP-43 immuno-staining signals (6-nm gold particles) were detected in close proximity to LonP1 immuno-staining signals (15-nm gold particles) (marked by the arrowheads in Fig 6C).

**Fig 7:**
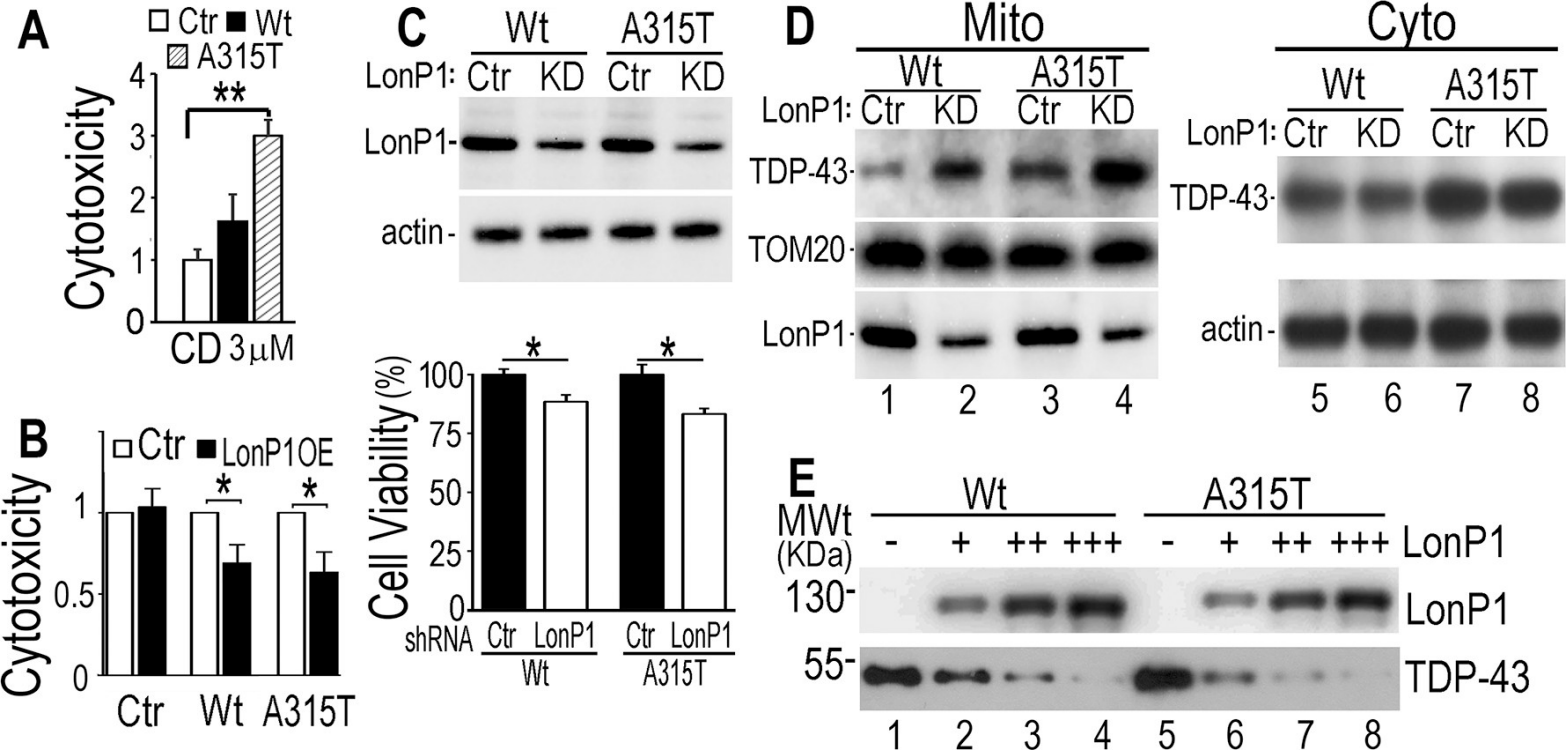
LonP1 degrades TDP-43 in cultured cells and *in vitro*. Treatment by the LonP1 inhibitor enhances TDP-43 induced cytotoxicity. (**A, B**) CD treatment enhanced, whereas LonP1 overexpression suppressed, TDP-43 induced cytotoxicity. Data in panels A and B represent 4 independent experiments (*: *P*<0.05, **: *P*<0.01; Student’s *t*-test). **(C, D) Down-regulating LonP1 increases TDP-43 induced cytotoxicity.** Control or shRNA specifically targeting LonP1 was transduced into inducible TDP-43 stable cells expressing either Wt or A315T-mutant TDP-43. Cell viability was measured 48 hr following induction of TDP-43. Down-regulating LonP1 significantly reduced viability of cells expressing either Wt or A315T-mutant TDP-43. Fractionation experiments revealed that mitochondrial TDP-43 levels were increased in cells in which LonP1 level was down-regulated (D). (**E**) TDP-43 protein was degraded by the purified recombinant LonP1 protein in an *in vitro* degradation assay. Wt or A315T-mutant TDP-43 protein was purified from the inducible HEK293 cells and incubated with increasing concentrations (0.5, 1.5 and 2.5 uM) of purified recombinant LonP1 (see Methods). The reaction products were analyzed by Western blotting using specific antibodies against TDP-43 and LonP1. Data in panels C-E represent three independent experiments.

A number of studies suggest the roles of proteasome and autophagy in degradation of TDP-43 [50–57]. We then tested the effects of a proteasome inhibitor (MG132, MG) and an autophagy inhibitor (3-methyladenine, MA), and compared them with that of a LonP1 inhibitor [2-cyano-3,12-dioxooleana-1,9-dien-28-oicacid, CDDO (CD) [58] ] in the inducible TDP-43 expressing cells. Interestingly, neither the proteasome inhibitor (MG) nor the autophagy inhibitor (MA) had an effect on cell viability following induction of TDP-43 expression, whereas the LonP1 inhibitor (CD) specifically reduced the viability of cells expressing either Wt or A315T-mutant TDP-43 and enhanced TDP-43 cytotoxicity (see S5A and S5B Fig). At the concentrations used, none of these drugs affected viability of the control cells, indicating that the effect of the LonP1 inhibitor was specifically associated with TDP-43 expression (Fig 7A; S5A and S5B Fig).

We next examined whether increasing LonP1 expression suppressed TDP-43 cytotoxicity. Control (Ctr) or TDP-43 expressing cells were transfected with a vector control (-) or a LonP1-expressing plasmid (+) 24hr before Tet-induction; and cells were examined 36 hr post-induction. Increased LonP1 expression suppressed TDP-43 induced cytotoxicity (Fig 7B). Quantification of Western blotting (WB) signals showed a ~2-fold increase in LonP1 expression, as normalized by actin levels. The total TDP-43 levels did not show significant changes (see S5C Fig), which is not unexpected because TDP-43 protein is predominantly nuclear, although it is the cytoplasmic/mitochondrial levels of TDP-43 that are correlated with neurotoxicity, as shown by published studies including ours [28,59].

We further tested whether down-regulating LonP1 altered TDP-43 induced cytotoxicity. TDP-43 inducible stable cells were transduced with a vector control virus (Ctr) or a lentivirus expressing shRNA specifically targeting LonP1 (KD) that reduced the LonP1 protein level by ~50%. LonP1 knockdown (KD) significantly reduced the viability in cells expressing TDP-43 (Fig 7C). Fractionation experiments demonstrated that LonP1 down-regulation led to an increase in mitochondrial TDP-43 protein level in these cells although the cytosolic levels of TDP-43 were not dramatically affected (Fig 7D), indicating that LonP1 decreases the mitochondrial TDP-43 protein level. To test whether TDP-43 could be directly degraded by LonP1, we established an *in vitro* protein degradation assay using purified recombinant LonP1 protein. Our data demonstrated that purified Wt or A315T TDP-43 protein was degraded by the purified recombinant LonP1 protein in a manner dependent on LonP1 concentrations (Fig 7E) and dependent on ATP (see S5D Fig).

A number of other mitochondrial proteases are involved in mitochondrial proteostasis. The mRNA level of CG5045, the *Drosophila* homolog of ClpP, was also increased in transgenic TDP-43 flies (see Fig 5B). We thus examined if TDP-43 also interacted with ClpP. However, no detectable interaction between ClpP and TDP-43 was observed in a co-immunoprecipitation assay (supplemental S6A Fig). Consistently, down-regulation of ClpP did not affect the mitochondrial TDP-43 level, as shown by WB analyses of purified mitochondria from cells following ClpP knockdown (supplemental S6B Fig). Together, these data show that LonP1 reduces TDP-43-induced cytotoxicity, possibly by degrading mitochondrial TDP-43 protein.

### LonP1 protects against TDP-43-induced mitochondrial damage and neurodegeneration *in vivo*

To investigate whether altering Lon expression *in vivo* would modify neurodegeneration induced by TDP-43, we obtained fly lines over-expressing the *Drosophila* LonP1 ortholog, Lon, or expressing specific siRNA against Lon. Only one fly line overexpressing Lon was available, and it showed ~2-fold increase in Lon mRNA expression compared with control flies when the Elav-Gal4 driver was used (see S7A Fig). However, over-expressing Lon by itself in control flies led to retinal degeneration. This prevented us from testing the effect of over-expressing Lon in TDP-43 flies.

**Fig 8:**
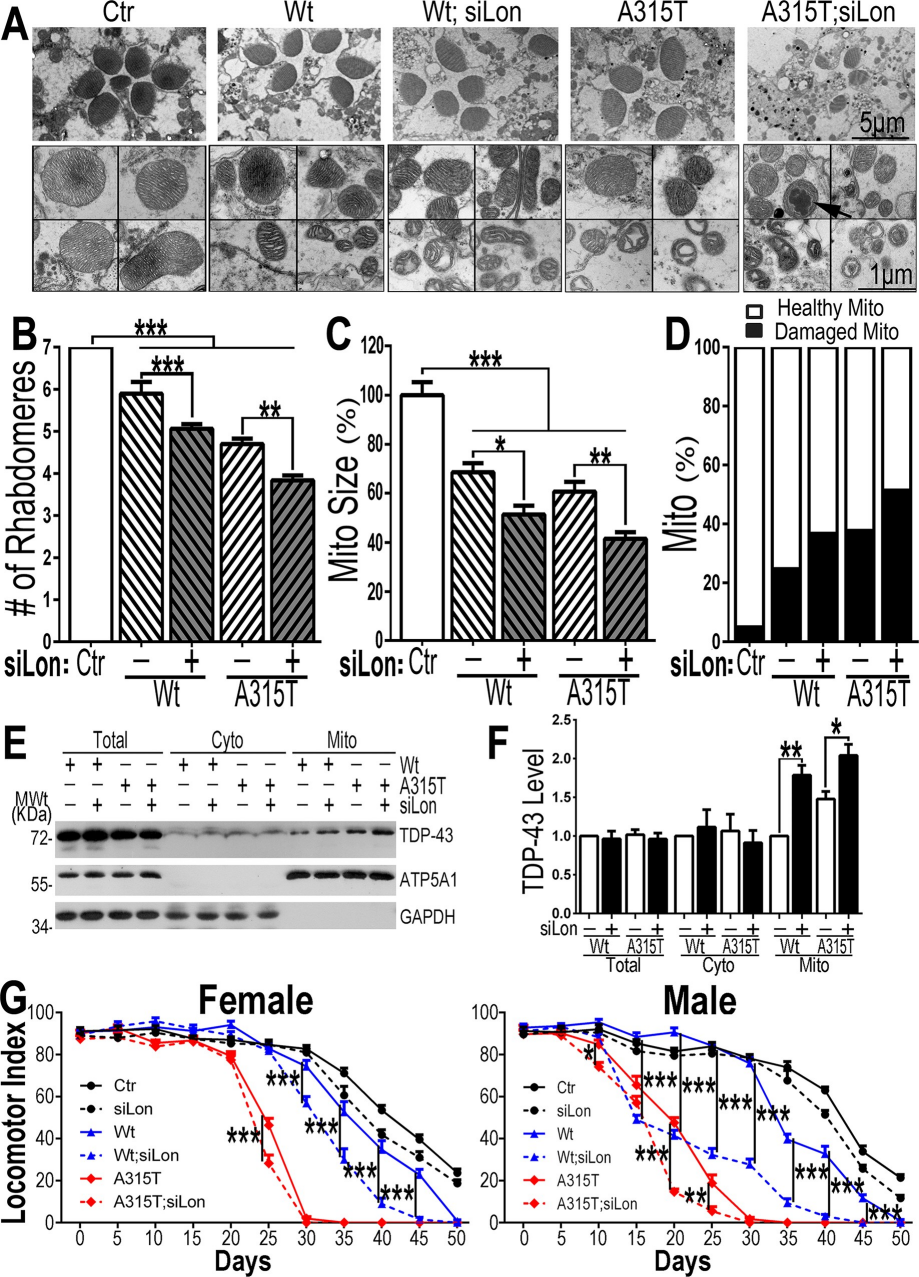
Down-regulation of Lon exacerbated retinal degeneration, mitochondrial damage and locomotor deficits in adult flies expressing TDP-43 protein. **(A**). TEM revealed that down-regulation of Lon exacerbated retinal degeneration and mitochondrial damage induced by expression of Wt or A315T-mutant TDP-43 (Day 20 post-induction). Top panels reveal the morphology of rhabdomeres in each fly group, whereas the higher magnification EM images in the lower panels show mitochondrial morphology in the corresponding groups (6 flies in each group were used). Mitochondrial damage, including cristae swelling and fragmentation, was more severe when Lon was down-regulated. The arrow points to an electron-dense aggregate (see **S10 Fig** for more examples). (**B**) Quantification of the number of rhabdomeres in each group of flies as indicated in (A). More than 70 ommatidia from 6 flies in each group (ommatidial number in each group, Ctr: 81; Wt: 76; Wt+siLon: 99; A315T: 79; A315T+siLon: 74 respectively) were quantified for each group. (**C,D**) Quantitative analyses indicate that the expression of Wt or A315T-mutant TDP-43 led to a significant reduction in mitochondrial size (C), and that down-regulation of Lon increased the percentage of damaged mitochondria (D). In flies expressing Wt TDP-43, ~24.6% mitochondria showed obvious fragmentation or swollen cristae, whereas the percentage of damaged mitochondria was increased to 36.6% when Lon was knocked-down in these flies. On the other hand, down-regulating Lon in flies expressing the A315T-mutant TDP-43 expressing flies increased the percentage of damaged mitochondria from 37.7% to 51.2%. In panel C, more than 100 mitochondria (Ctr: 125; Wt: 123; Wt+siLon: 117; A315T: 138; A315T+siLon: 124 respectively) from 6 flies in each group were quantified. In panel D, more than 400 mitochondria (Ctr: 431; Wt: 414; Wt+siLon: 445; A315T: 494; A315T+siLon: 511 respectively) were quantified for each group. (**E, F**) Biochemical fractionation experiments revealed an increase in the mitochondrial TDP-43 levels. Mitochondria were purified from the eyes of the corresponding groups of flies and analyzed using WB with specific antibodies together with the total cell lysates (Total) and cytoplasmic fractions (Cyto). (**F**) Quantification of mitochondrial TDP-43 levels indicates that down-regulating Lon led to an accumulation of TDP-43 in mitochondria in flies expressing either Wt or A315T-mutant TDP-43. Data represent three independent experiments. (**G**) The locomotor index was measured in adult flies at different time points following induction of expression of Wt or A315T-mutant TDP-43 under the Elav-Gal4/Tub-Gal80^ts^ driver. Expression of TDP-43 in these flies led to progressive locomotor deficits, with A315T-mutant TDP-43 expressing flies showing a more severe phenotype. Down-regulation of Lon led to an earlier onset and more severe locomotor deficits in flies expressing TDP-43. The exacerbating effect of down-regulating Lon seemed to be more pronounced in male flies. More than 100 flies were analyzed in each group (precise fly numbers of each group in Supplementary Information). Data represent two independent experiments. Data in panels B, C, F and G were analyzed using a one-way ANOVA with Bonferroni post hoc test (*:*P*<0.05; **: *P* <0.01; ***: *P*<0.001). Fly genotypes for panels A-D: **Ctr**: GMR-Gal4/Tub-Gal80^ts^ /UAS-RFP; **Wt:** GMR-Gal4/Tub-Gal80^ts^ /UAS-Wt-TDP43; **Wt; siLon**: GMR-Gal4/Tub-Gal80^ts^ /UAS-Wt-TDP-43/UAS-siLon; **A315T**: GMR-Gal4/Tub-Gal80^ts^ /UAS-A315T-TDP-43; **A315T;siLon**: GMR-Gal4/Tub-Gal80^ts^ /UAS-A315T-TDP-43/UAS-siLon. Fly genotypes for panels E-F:**Wt**: GMR-Gal4/UAS-Wt-TDP43; **Wt;siLon**: GMR-Gal4/UAS-Wt-TDP-43/UAS-siLon; **A315T:** GMR-Gal4/UAS-A315T-TDP-43; **A315T;siLon**: GMR-Gal4/UAS-A315T-TDP-43/UAS-siLon. Fly genotypes for panel G: **Ctr**: Elav-Gal4/Tub-Gal80^ts^ /UAS-RFP; **siLon:** Elav-Gal4/Tub-Gal80^ts^ /UAS-RFP/UAS-siLon; **Wt**: Elav-Gal4/Tub-Gal80^ts^ /UAS-Wt-TDP-43; **Wt; siLon**: Elav-Gal4/Tub-Gal80^ts^ /UAS-Wt-TDP-43/UAS-siLon; **A315T**: Elav-Gal4/Tub-Gal80^ts^ /UAS-A315T-TDP-43; **A315T; siLon**: Elav-Gal4/Tub-Gal80^ts^ /UAS-A315T-TDP-43/UAS-siLon.

On the other hand, two siLon fly lines were obtained, #1 and #2, which reduced Lon expression to ~30% and ~60%, respectively, of that in the control flies (see S7A Fig). Down-regulating Lon expression by itself in control flies did not cause detectable phenotypes. The siLon#1 fly line showed more robust down-regulation efficiency and was thus used in subsequent experiments. We then crossed siLon flies with TDP-43 transgenic flies and examined retinal degeneration and locomotor function in adult flies expressing TDP-43 in photoreceptors or in all neurons respectively. Using the GMR-Gal4/tubulin-Gal80^ts^ driver, we monitored the progression of retinal degeneration during the adult stage following induction of TDP-43 expression by pulses of heat shock. Retinal degeneration was examined using TEM. By day 20 following TDP-43 induction, flies expressing TDP-43 exhibited profound retinal degeneration. The control flies showed normal photoreceptor organization, and heat shock per se did not affect photoreceptor development or maintenance as previously reported [60]. In contrast, retinae in flies expressing TDP-43 showed ommatidial disorganization with a clear reduction in rhabdomere numbers. The average number of rhabdomeres in flies expressing Wt or A315T-mutant TDP-43 was 6 or 5 respectively, as compared with 7 in the control flies (Fig 8A, Fig 8B). In flies expressing Wt or A315T-mutant TDP-43, down-regulating Lon expression exacerbated retinal degeneration, reducing the average rhabdomere number to 5 (Wt; siLon) or 4 (A315T; siLon), respectively (Fig 8A and 8B).

Biochemical fractionation experiments indicate that down-regulating Lon in these flies led to an increase in the mitochondrial TDP-43 level, although there was no significant increase of the TDP-43 levels in the total cell lysates or in the cytosol (see Fig 8E, Fig 8F; S7B and S7C Fig). We further examined solubility of mitochondrial TDP-43 in these flies following sequential extraction using NP-40, SDS and urea. Down-regulating Lon increased the mitochondrial TDP43 protein level, especially the NP-40 soluble fraction in the Wt TDP-43 group and SDS-resistant/Urea-soluble fraction (in the urea lanes) in the A315T-mutant TDP43 group (see S8 Fig). Importantly, Lon knockdown in TDP-43 expressing flies exacerbated mitochondrial damage, with a further reduction in mitochondrial size and an increase in the percentage of damaged mitochondria in the retinae (Fig 8C, Fig 8D), although knock-down Lon by itself in the control flies did not affect rhabdomere or mitochondrial morphology (see S9 Fig). These results demonstrate that mitochondrial TDP-43 accumulation correlates with TDP-43-induced mitochondrial damage and neurodegeneration. Intriguingly, electron-dense aggregate-like structures were detected inside mitochondria in A315T; siLon flies (Fig 8A, marked by a black arrow in the lower panel in the “A315T; siLon” panel; also see S10 Fig, marked by an arrow). These electron-dense aggregate-like structures were not detected in any other groups of flies. Molecular characterization of these electron-dense aggregate-like structures awaits further studies in the future.

We also examined the effects of down-regulating Lon on the locomotor function of the flies expressing TDP-43 under the Elav-Gal4/tubulin-Gal80^ts^ driver. Flies expressing TDP-43 showed progressive locomotor defects following induction of TDP-43 expression, with flies expressing A315T-mutant TDP-43 showing more severe defects. Down-regulating Lon expression in flies expressing Wt or A315T-mutant TDP-43 exacerbated the locomotor defects induced by TDP-43 (Fig 8G). In flies expressing Wt TDP-43, Lon knockdown significantly reduced locomotor function by day 15 onward in males and day 30 onward in females. In flies expressing A315T-mutant TDP-43, Lon knockdown significantly reduced locomotor function by day 10 onward in males and day 25 onward in females. The exacerbating effect of Lon down-regulation appeared more pronounced in males than in females. The onset of locomotor deficits was advanced in both females and males expressing Wt TDP-43. By day 40 post-induction of TDP-43 expression, in flies expressing Wt TDP-43 the locomotor index was >30, whereas down-regulating Lon in Wt TDP-43 flies led to a complete loss of locomotor function in both females and males (Fig 8G). These results show that Lon plays a protective role against TDP-43 induced neurodegeneration in these flies, especially during the early stage of the disease. Together, our data indicate that mitochondrial damage contributes to TDP-43-induced neurodegeneration.

## Discussion

TDP-43 is a multi-functional RNA/DNA binding protein involved in multiple processes of gene regulation, from chromatin remodeling, DNA stability to RNA processing, including microRNA biogenesis, transcriptional and splicing regulation, mRNA trafficking as well as mRNA stability regulation [3,4,61]. Over a decade ago, TDP-43 was identified as a characteristic protein in the inclusion bodies of tissues from patients affected by TDP-43 proteinopathy, including ALS-TDP and FTLD-TDP [1,62]. Since then, a large number of mutations in the TDP-43 gene have been identified in ALS patients, whereas dysregulation of TDP-43 gene expression or its function has been found in patients affected by FTLD and other neurodegenerative disorders [4,63,64].

Several groups have reported mitochondrial abnormalities in different models for TDP-43 proteinopathy, including abnormal mitochondrial clustering [24,26], and a shift in dynamics toward mitochondrial fragmentation [20,22,23,25]. A recent study reported the accumulation of TDP-43 in mitochondria in TDP-43 proteinopathy brain samples [28]. Of these studies, only one reported ultrastructural changes of mitochondria in mice expressing A315T-mutant TDP-43 [20]. However, it was not clear how widespread this damage was. There has not been, to our knowledge, a systematic morphological characterization of mitochondria in patient samples nor in TDP-43 proteinopathy model systems. Our study builds on these previous results by systematically and quantitatively examining TDP-43 induced mitochondrial damage using EM and other methods across different model systems and in patient samples. Our EM analyses clearly show that mitochondria frequently exhibited severe morphological impairment in TDP-43 proteinopathy patient samples and that such mitochondrial morphological changes are consistently detected across cellular and animal models of TDP-43 proteinopathy (Fig 1, Fig 2, Fig 4 and Fig 8). Interestingly, swollen mitochondrial cristae detected in the TDP-43 expressing cells and animals, and in patient samples, are reminiscent of the mitochondrial abnormality in mice expressing SOD1 mutant [65].

Recent studies indicate that cristae morphology determines the assembly and stability of respiratory chain super-complexes, and affects mitochondrial function [66,67]. It is not surprising that mitochondrial cristae are affected in a range of diseases, including neurodegenerative disorders. It has been reported that mitochondrial cristae are disrupted in Alzheimer's disease, showing concentric or parallel stacks [68,69]. A previous study from our group revealed that mitochondria in FTLD-FUS brain tissues showed a marked loss or disruption of cristae, with frequent detection of mitochondria in an “onion-like” deformed shape [70]. Data presented in this study demonstrate that vesicular or swollen mitochondrial cristae are a prominent feature not only in our cellular or animal models, but also in patient samples of TDP-43 proteinopathy (Fig 1, Fig 2 and Fig 4). Our results together with previous studies support the notion that mitochondrial impairment is a common pathogenic contributor to neurodegenerative diseases, and that distinct ultrastructural changes in mitochondria may reflect different mechanisms leading to mitochondrial damage.

Consistent with the morphological changes that we observed, mitochondrial membrane potential and mitochondrial ATP synthesis were reduced upon induction of TDP-43 expression (Fig 2). Interestingly, TDP-43 expression suppressed the activity of mitochondrial complex I, and to a lesser extent, complex IV, without affecting complexes II, III or V (Fig 2). The effect of TDP-43 on ATP synthesis and respiratory complexes has been examined in previous studies, but with discrepant results [23,27–29,71]. Onesto and colleagues observed no change in the total ATP level and reduced mitochondrial membrane potential in fibroblasts from ALS-TDP patients (carrying the A382T mutation), consistent with our results; however, they observed no differences in mitochondrial complex activities. Kawamata and colleagues, on the other hand, reported that there were no mitochondrial bioenergetic defects in fibroblasts or transgenic mice expressing TDP-43 mutants, although mitochondrial calcium handling seemed to be affected [29]. In contrast, Wang and colleagues observed a decrease in ATP synthesis and a decrease in relative levels and activity in complex I from fibroblasts from ALS-TDP patients and HEK293 cells transiently overexpressing wild-type or three ALS-mutants of TDP-43; however, they did not observe changes in the other complexes. Two groups provided evidence for mitochondrial dysfunction, including reduced mitochondrial respiration and ATP synthesis, in NSC-34 cells expressing ALS-mutant TDP-43 [27,71]. Further studies are necessary to resolve the discrepancy in these studies.

Our data presented here show that TDP-43 increases mitochondrial ROS production both *in vitro* and *in vivo* (Fig 2; S4 Fig). Mitochondrion is a major site for ROS production, and excessive ROS accumulation can further damage mitochondria [43,72,73]. Although there were no detectable effects of TDP-43 on ROS production in cultured fibroblasts in the previous study [23], data from our cellular model show a clear increase in mitochondrial ROS production induced by TDP-43 (Fig 2). Furthermore, TDP-43 expression in fly motor neurons significantly increased mitochondrial ROS levels *in vivo* (S4 Fig).

It is interesting to note that the electron-dense TDP-43 positive aggregates detected inside mitochondria in TDP-43 proteinopathy patient brain samples (Fig 1D) are reminiscent of the EM findings in lymphoblasts expressing LonP1 mutations of patients affected by cerebral, ocular, dental, auricular, skeletal (CODAS) syndrome [74]. The mitochondrial abnormalities reported in these CODAS patients are similar to those detected in our TDP-43 proteinopathy patient samples, including swollen intra- or intercristal compartments, swollen or vesicular cristae and intra-mitochondrial aggregate-like structures (see Fig 1) [74]. Intriguingly, similar intra-mitochondrial aggregates were detected in flies expressing A315T-mutant TDP-43 only when *Drosophila* LonP1 homolog, Lon, was down-regulated (see S9 Fig). Given that LonP1 is an ATP-dependent mitochondrial protease [49,74], and that mitochondrial ATP synthesis is suppressed by TDP-43, it is possible that reduced mitochondrial ATP synthesis might affect proteolytic activity of LonP1, resulting in further TDP-43 accumulation within mitochondria as the disease progresses and eventually leading to irreversible mitochondrial damage and the demise of affected neurons.

Our data from both mammalian cells and transgenic flies show that TDP-43 expression elicits UPR^mt^, a program that is evolutionarily conserved from nematodes to mammals. UPR^mt^ induces expression of mitochondrial chaperones to assist in proper protein folding and proteases to promote clearance of misfolded proteins [30–32,75]. A variety of mitochondrial stresses induce UPR^mt^, including accumulation of misfolded proteins, depletion of mitochondrial DNA, ROS overload, perturbation of OXPHOS or mitochondrial translation, and disruption of the balance between mitochondrial- and nuclear-encoded proteins [30–32,76,77]. UPR^mt^ has been reported in Parkinson's disease, Alzheimer’s disease and ALS-SOD1 [33–35]. UPR^mt^ activation detected in our cellular and animal models for TDP-43 proteinopathy could be the result of the combined effects of TDP-43, including mitochondrial accumulation of TDP-43 protein, increased ROS production, decreased membrane potential, impaired respiratory chain function and decreased mitochondrial ATP synthesis. To our knowledge, there were no previous reports of UPR^mt^ in TDP-43 proteinopathy.

Consistent with qPCR results from cellular and fly models, the LonP1 protein level was up-regulated in a fraction of patients affected by TDP-43 proteinopathy (Fig 5). Our data show that LonP1 interacts with TDP-43 and that purified LonP1 degrades TDP-43 (Fig 6 and Fig 7). More importantly, inhibition or down-regulation of Lon led to increased mitochondrial TDP-43 accumulation and exacerbated mitochondrial damage and neurodegeneration phenotype *in vivo* (Fig 8). It is conceivable that balanced protein synthesis and degradation of TDP-43 is critical for ensuring proper function of TDP-43 in the nucleus, cytosol and mitochondria. Recently, a new mechanism of mitochondria-mediated proteolysis, known as “mitochondria as guardian in cytosol (MAGIC)”, was reported for degrading mis-foled proteins [78]. By MAGIC, cytosolic proteins prone to aggregation can be imported into mitochondria for degradation by mitochondria proteases in yeast and human cells, and *PIM1* (encoding yeast Lon protease) is a major player in this process [78]. The complete machinery for MAGIC remains to be defined. Further studies are necessary to determine whether MAGIC is a major mechanism in mammalian proteostasis.

**Fig 9:**
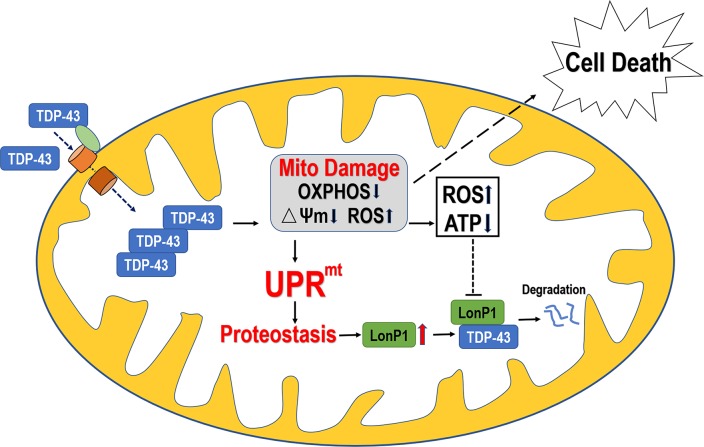
A working model to illustrate that accumulation of mitochondrial TDP-43 leads to mitochondrial damage and triggers UPR^mito^.

Together, our data led to a working model for the role of mitochondrial degradation of TDP-43 in the pathogenesis of TDP-43 proteinopathy (Fig 9). Under physiological conditions, TDP-43 is predominantly nuclear, although it shuttles between the nucleus and cytoplasm, with a small amount of TDP-43 transported into mitochondria. When TDP-43 mutations occur, or under certain cellular stresses, the mitochondrial TDP-43 level is increased. Excessive mitochondrial TDP-43 accumulation results in mitochondrial impairment, manifesting as mitochondrial membrane potential loss, mitochondrial ROS increase, and reduced mitochondrial ATP synthesis. Such TDP-43-induced mitochondrial damage triggers UPR^mt^, allowing the cell to initiate a series of responses to regain mitochondrial proteostasis by up-regulating mitochondrial proteases, including LonP1. It is likely at this early stage, before mitochondrial damage becomes irreparable, that mitochondrial stress responses enable the cell to reverse mitochondrial dysfunction. However, as the disease progresses, chronic cellular stresses lead to the excessive accumulation of TDP-43 in mitochondria, inducing irreversible mitochondrial damage. For example, persistent increase in the ROS level and severe reduction in ATP synthesis may result in a vicious cycle of suppression of LonP1 proteolytic activity and further accumulation of mitochondrial TDP-43 in spite of an increased protein level of LonP1, culminating in activation of cell death program(s). Data from our animal model and patient samples, together with our *in vitro* findings, support the notion that LonP1 may provide a protective mechanism against TDP-43 mediated neurotoxicity. It is noted that the time courses of TDP-43-induced UPR^mt^ gene activation showed differences in male and female flies (Fig 5B). Intriguingly, the exacerbation of locomotor deficits by Lon knockdown appeared to be more pronounced in male flies (Fig 8G). This is consistent with a previous report that expression patterns of Lon protein isoforms were different between male and female flies and that Lon was required for gender-specific responses to oxidative stress [79]. The mechanisms underlying such gender-specific stress responses remain to be elucidated. Further work is necessary to determine whether the gender-specific response(s) play a significant role in humans against neurodegeneration.

Since the discovery of TDP-43-containing inclusion bodies in ALS and FTLD patient samples, intense efforts have been made to identify proteases capable of degrading TDP-43. A number of elegant studies have proposed possible involvement of different proteases in degrading TDP-43, including caspases, calpain and asparaginyl endopeptidase [56,80–86]. None of the previously identified proteases have been shown to protect against TDP-43 induced neurotoxicity *in vivo*. Our biochemical experiments show that the endogenous TDP-43 and LonP1 interact with each other and that TDP-43 is degraded by the purified recombinant LonP1. Down-regulating LonP1 drosophila homolog, Lon, exacerbates TDP-43 induced mitochondrial damage and neurodegeneration. Together, these data provide previously unknown evidence that the mitochondrial protease LonP1 can protect against TDP-43 induced neurodegeneration *in vivo*. It will be interesting to investigate in the future whether genetic or epigenetic alterations that affect the expression or function of the human LonP1 gene may influence the onset or progression of TDP-43 proteinopathy. Our study suggests that improving mitochondrial function and reducing mitochondrial damage may provide therapeutic potential for patients affected by TDP-43 proteinopathy.

## Materials and methods

### Ethics statement

De-identified postmortem human brain samples from autopsied tissues at the Neuropathology Core of the Cognitive Neurology & Alzheimer's Disease Center at Northwestern University were used following NIH and institutional guidelines. There was no research involving human subjects in this study. All animal studies were performed in accordance with national and institutional guidelines.

### Cell cultures and transfection

HEK293 cells were cultured (37°C, 5% CO2) in DMEM (Gibco), supplemented with 10% FBS (Gibco) and transfected as previously described [70]. HEK293-based T-Rex293 cells (Invitrogen) were transfected with pcDNA4 TO/myc-His plasmids (Invitrogen) expressing either Wt, or A315T-mutant TDP-43 following the manufacturer’s manual. Control cells were transfected with an empty pcDNA4 vector. Individual clones of cells stably expressing TDP-43 were obtained following selection in zeocin (400 μg/mL). To induce TDP-43 expression, tetracycline (0.5μg/mL; unless specified otherwise) was added to the culture medium, and cells were cultured for different periods of time at 37°C until harvesting. Western blotting was used to confirm induction of TDP-43 protein expression.

### Fly strains and antibodies

Transgenic flies expressing the human TDP-43 (Wt or A315T-mutant) were described previously [40,41,87]. GMR-Gal4, OK371-Gal4, Elav-Gal4 and UAS-Lon-RNAi lines were obtained from the Bloomington Drosophila Stock Center (BDSC). Another UAS-Lon-RNAi fly line was obtained from the Vienna Drosophila Resource Center (VDRC). UAS-dLonOE was from the Kyoto Stock Center. The Tubulin-Gal80^ts^ (Tub-Gal80^ts^) line was kindly provided by Dr. A. Guo (IBP, CAS) [48]. The UAS-mito-roGFP2-Grx1 fly lines were kindly provided by Dr. T. Dick [47].

For flies under the Elav-Gal4/Tub-Gal80^ts^-driver or GMR-Gal4/Tub-Gal80^ts^-driver, parental flies were crossed and cultured at 18°C, young flies after eclosion were transferred to 28°C for 4 hr every day to induce TDP-43 expression. Other flies were all cultured at 25°C. All flies were raised in standard fly food, 50% relative humidity, and 12hr-12hr light-dark cycles as described previously [41,70,87].

Antibodies used in this study include polyclonal rabbit-antibodies against TDP-43, ATP5A1, LonP1, HSPA9, ClpP, TOM20 and IMMT (ProteinTech Group Inc), as well as mouse monoclonal antibodies, anti-actin (ProteinTech Group Inc), anti-HSP60 (BD Biosciences) and anti-GAPDH (CWBIO). Rat-anti-dElav antibody is a kind gift from Dr. A. Guo.

### Transmission electron microscopy and immuno-electron microscopy

Brain samples were evaluated for atrophy and for pathology by hematoxylin-eosin staining and immunostaining using corresponding antibodies, as previously described [40]. The brain tissue samples were fixed in 2.5% glutaraldehyde (GA, Electron Microscopy Sciences) for 2–3 hr at room temperature, after washing with PBS and fixation in 1% OsO4 buffer for 2 hr, the samples were dehydrated with graded ethanol solutions, and then embedded in Epon812 resin (SPI). Ultrathin sections (70 nm) were stained with 2% uranyl acetate for 30 minutes and then lead citrate for 10 minutes before imaging using an electron microscope (TecnaiTM Spirit, FEI).

For fly EM samples, fly heads were collected at day 3, fixed in 4% paraformaldehyde (PFA, Electron Microscopy Sciences) and 2.5% GA overnight at 4°C. For HEK293 cells, cells were rinsed with PBS and then fixed in 2.5%GA overnight at 4°C. TEM sections were prepared following protocols as described previously [88]. Fly heads and cells were then treated in the same manner as the brain tissues described above and sectioned on a Leica EM UC6/FC6 Ultramicrotome. After sections were transferred to copper grids, counter staining was performed with uranyl acetate and lead acetate before EM imaging.

Immuno-EM was carried out following our published protocol [70]. Briefly, samples were fixed in 2% PFA and 0.2% GA overnight. After rinsing with PBS, samples were embedded in 12% gelatin, dehydrated in 2.3M sucrose, subjected to ultrathin sectioning (70 nm) and then mounted on copper grids. After an additional rinse with PBS (with 1% BSA and 0.15% Glycine), samples were blocked in 5% goat serum (Electron Microscopy Sciences, EMS) for 30 minutes. Immunostaining was performed, incubating with primary antibodies for 2 hr followed by immunogold labeled secondary antibodies (EMS) for 1.5 hr. Following rinses with PBS, samples were re-fixed with 2.5% GA for 10 minutes and stained with 4% Uranyl acetate for 5 minutes, and imaged under a FEI TECNAI SPIRIT electron microscope.

### Measurement of mitochondrial membrane potential by JC1

Mitochondrial membrane potential was measured in inducible TDP-43 cell lines using the mitochondrial dye JC1 (Invitrogen) following a published protocol [89]. Briefly, 48 hr before assay, inducible stable cells expressing the control vector or TDP-43 were seeded in 6-well plates. Tetracycline (1μg/mL) was added to induce TDP-43 expression for 0, 24 or 36 hr. Cells were detached using Trypsin-EDTA, rinsed in cold PBS and then stained using JC1 (5uM) for 20 minutes at 37°C. Following staining, cells were measured using flow cytometry (BD FACS Calibur) and were analyzed by FlowJo software. Data were obtained from four independent experiments. More than 20,000 cells were measured per group in each experiment.

### Measurement of mitochondrial ROS levels in fly motor neurons

Image acquisition and analyses of mitochondrial ROS of larval VNC motor neurons were performed according to published protocols with slight modifications [47]. Briefly, OK371-Gal4/UAS-mito-roGFP2-Grx1 flies were crossed with female control or TDP-43 transgenic flies. Third instar wandering larvae were dissected in PBS containing 20mM N-ethyl maleimide (NEM) (Sigma-Aldrich), and incubated for 10 minutes. Larvae were then rinsed with PBS and then fixed with 4% PFA before mounting. Fixed larval ventral nerve chord (VNC) samples were imaged with a Leica SP8 confocal microscope equipped with a 40X oil immersion objective. Probe fluorescence was excited sequentially at 405 nm (reduced roGFP) and 488 nm (oxidized roGFP) (frame by frame) and detected at 500–530 nm. A ratio image was created by dividing a 405-nm image by the corresponding 488-nm image pixel-by-pixel, resulting in the ratio of reduced to oxidized roGFP. Images were processed and quantified using ImageJ.

### Measurement of the total cellular ATP levels

The total cellular ATP level was measured using a CellTiter-Glo Luminescent Assay (Promega) according to the manufacturer’s instruction. Briefly, 48 hr before assay, the control, Wt or ALS-mutant TDP-43 stale HEK293 cells were seeded in 96-well plates. One μg/mL tetracycline was added to induce TDP-43 expression for 0, 12, 24, or 36 hr. Following removal of the culture media and cell lysis, reaction mixtures were transferred to another opaque 96-well plate to measure luminescence. Luminescent signal values were normalized by the protein amount in each group to determine the total cellular ATP levels.

### Mitochondrial purification

Mitochondrial isolation was performed according to published protocols with minor modifications [38,70]. Briefly, stable TDP-43-expressing HEK293 cells were suspended in isolation butter [0.22M mannitol, 0.07M sucrose, 20mM HEPES (pH 7.2), 1mM EGTA], homogenized with a Glass/Teflon Potter Elvehjem homogenizer (Bellco Glass Inc) and then fractionated by sequential centrifugation. Pellets (the mitochondrial fraction) were washed twice with wash buffer (0.25M sucrose, 50mM HEPES, 1mM EGTA, pH7.4) and were then resuspended in the same buffer. The protein amount was determined by the BCA protein assay (Pierce).

Fly mitochondrial purification was performed according to a published protocol with minor changes [90]. Sixty fly heads were collected under a microscope and were transferred into a Glass-Teflon Dounce homogenizer containing 500 μL of cold isolation buffer (225 mM Mannitol, 75 mM Sucrose, 10 mM MOPS and 1 mM EDTA, 2.5 mg/mL BSA) and homogenized on ice for 20 strokes. The homogenate was transferred to a 1.5 ml tube for centrifugation at 600 g for 10 min at 4°C. The supernatant was centrifuged at 8,000 g for 10 min at 4°C to enrich for mitochondria. Mitochondrial pellet was washed with 0.5 ml wash buffer (225 mM Mannitol, 75 mM Sucrose, 10 mM KCl, 10 mM Tris-HCl and 5 mM KH_2_PO4) and were then resuspended in the same buffer.

### Mitochondrial ATP synthesis assay

Mitochondrial ATP synthesis was measured using a published protocol with minor modifications [39]. Briefly, equal amounts (30μg) of purified mitochondria were incubated with reaction substrates (0.15mM P1, P5-di (adenosine) pentaphosphate; 2mM malate; 2mM pyruvate; 0.1mM ADP) with or without oligomycin at 37°C for 5 minutes. Reaction mixtures were stopped by adding boiling stop buffer (100mM Tris-HCl, 4mM EDTA, pH 7.4) and then an equal amount of CellTiter-Glo reagent (Promega) was added to measure ATP using a microplate reader. Mitochondrial ATP synthesis was quantified by subtracting the ATP content in the presence of oligomycin from the ATP content in the absence of oligomycin of the corresponding group.

### Measuring activities of mitochondrial respiratory complexes

Stable inducible HEK293 cells expressing either the vector control or TDP-43 (Wt or A315T-mutant) were established as described above. Mitochondria were purified from these cells 24h following induction with tetracycline (1μg/mL) using a published protocol [70]. Briefly, mitochondria were collected from the boundary between 23% and 40% percoll of gradient centrifugation. Mitochondrial respiratory chain complex activities were measured following the published protocols [39,91]. Briefly, 10 μg of mitochondria were applied to a 100μl reaction mixture containing 30 mM KPO_4_ pH7.2, 5mM MgCl_2_, 2.5 mg/mL BSA, 0.3 mM KCN, 0.13 mM NADH, 2 μg/mL antimycin A and 97.5 μM ubiquinone-1. The complex I specific activity was determined by the subtraction of the nonspecific activity in the presence of rotenone from the total NADH oxidase activity in the absence of rotenone. Complex II activity was measured in reaction mixture containing 30 mM KPO_4_ (pH7.2), 5 mM MgCl_2_, 2.5 mg/mL BSA, 0.3 mM KCN, 50 μM DCPIP, 20mM succinate, 2 μg/mL antimycin A and 65 μM decylubiquinone. The complex II specific activity was determined by subtracting the nonspecific activity in the presence of malonate from the total ubiquinone reductase activity in the absence of malonate. Complex III and IV activities were measured by reduction and oxidation of cytochrome C, respectively, monitoring OD_550_ respectively, as described previously [91]. Complex V activity was measured by subtracting non-specific activity in the presence of oligomycin following the published protocol [39].

### Mitochondrial ROS detection assay

Mitochondrial ROS level was measured as described previously [70]. Briefly, 48 hr before the assay, inducible stable cells expressing the control or TDP-43 were seeded in 6-well plates. Tetracycline (1μg/mL) was added to induce TDP-43 expression for 0, 24, 36hr, respectively. Cells were detached using Trypsin-EDTA, rinsed in cold PBS and then stained with mitoSOX-Red for 20 min at 37°C. After washes, cells were fixed with 4% paraformaldehyde for 20 minutes at room temperature. Cells were measured using flow cytometry (BD FACS ArialI) within 1 hr with analyses using the FlowJo software. Data were obtained from four independent experiments, with more than 20,000 cells were measured per group in each experiment.

### Cell death detection assay

Cell death was measured using an Annexin V-FITC Apoptosis Detection Kit I (BD) according to the manufacturer’s instructions. Briefly, 48 hr before the assay, inducible stable cells expressing the control or TDP-43 were seeded in 6-well plates. Tetracycline (1μg/mL) was added to induce TDP-43 expression for 0, 24 or 36 hr. Cells were detached by Trypsin-EDTA, rinsed in cold PBS and then stained with Annexin V-FITC and propidium iodide (PI) followed by immediate analyses (within 1 hr) using flow cytometry (BD FACS Calibur). Data were obtained from four independent experiments, and more than 20,000 cells were measured per group in each experiment.

### Cell viability and cytotoxicity assays

Cell viability and cytotoxicity were determined using a CytoTox-ONE Homogeneous Membrane Integrity kit following the manufacturer’s instructions (Promega). Briefly, the activity of lactate dehydrogenase (LDH) results in the generation of the fluorescent resorufin product, which was measured using a SPECTRAmax GEMINI XS (Molecular Device; excitation at 560 nm and emission at 590 nm). The cellular LDH activity quantifies the number of viable cells (cell viability); and the activity of LDH released in the culture media quantifies the number of non-viable cells that have lost membrane integrity (cytotoxicity).

### Purification of LonP1 and in vitro protein degradation assay

A cDNA encoding the human LonP1 protein (amino acid residues 115–959) was cloned into vector pET32M3C [a modified version of the pET32a vector (Novagen, 69015–3)], expressed as an N-terminal thioredoxin and 6XHis-tagged protein and purified from E. coli (Rosetta strain, Novagen) following the published protocol [92]. Purified human LonP1 was analyzed by SDS-PAGE followed by Coomassie Brilliant Blue staining and by immunoblotting using an anti-LonP1 antibody. Following Tet-induction (1μg/mL tetracycline) of the inducible HEK293 cells for 36hr, MycHis-tagged Wt or A315T-mutant TDP-43 protein was purified using Ni-Sepharose (GE Healthcare). Purified TDP-43 protein was incubated in a 30 μL in vitro degradation reaction system [20 mM Tris-HCl (pH8.0), 20 mM NaCl, 10 mM MgCl_2_, 1 mM DTT, 5 mM ATP] with different concentrations of purified LonP1 protein for 90 min at 37°C. The reaction products were analyzed by Western blotting using the corresponding specific antibodies to detect TDP-43 and LonP1 proteins.

### RNA Isolation and qRT-PCR

Total RNA was isolated from HEK293 cells or fly heads using TRizol reagent (Invitrogen) as described previously [70]. cDNA synthesis and qPCR were performed as described [30,70,79] using the corresponding primers (see S2 Table). HPRT-1 and Actin5C were used as reference genes for mammalian cells and fly tissues, respectively.

### Fly locomotor assays

The adult fly locomotor assay was carried out as described previously with minor modifications [41]. Briefly, flies were examined every 5 days with their locomotor index measured as the percentage of flies climbing above a 6-cm line in 15 seconds after they were tapped to the bottom of an empty vial. The experiment was repeated 10 times for each group.

### Mitochondrial protein solubility assay

The protein solubility was examined as described previously with minor modifications [40]. Briefly, 100 fly heads were collected for mitochondrial purification. 100 μg of the mitochondrial fractions were resuspended in 200 μL RIPA lysis buffer containing 0.5% NP-40, extracted for 20 minutes on ice and then centrifuged at 12,000 g to collect the supernatant as the NP-40-soluble fraction and the pellet. The NP-40-insoluble pellet was then resuspended and extracted in 200 μL RIPA buffer containing 2% SDS for 20 minutes on ice. Following centrifugation at 12,000 g, the supernatant was collected as the SDS-soluble fraction. The SDS-insoluble pellet was then resuspended and extracted in 100 μL RIPA buffer containing 8 M urea for 20 minutes on ice. Following centrifugation at 12,000 g, the supernatant was collected as the urea-soluble fraction. All fractions were then subjected to Western blotting analysis.

### Statistical analyses

Data were collected in Excel (Microsoft) and analyzed using GraphPad Prism 6 unless specified otherwise. Differences between two groups were analyzed using a Student’s *t*-test. Multiple group comparisons were performed using a one-way or two-way analysis of variance (ANOVA) followed by post-hoc tests. The bar graphs with error bars represent mean ± standard error of the mean (SEM). Significance is indicated by asterisks: *, P < 0.05; **, P< 0.01; ***, P< 0.001.

## Supporting information

S1 Table Pathological and clinical diagnoses of subjects whose tissue samples were used in this study. All samples used were sequenced and confirmed that there were no mutations in known genes associated with ALS or FTLD, including TDP-43, FUS, C9orf72, GRN, SOD1 and MAPT, as reported previously [40]. Age, gender, Post-mortem interval (PMI; hours), together with pathological and clinical diagnoses, are included.
(DOCX)

S2 Table Primers used in qPCR experiments. (DOCX)

S1 Fig TDP-43 localizes to mitochondria and induces mitochondrial damage in HEK293 cells. (**A**) Western blotting experiments revealed that the Myc-tagged TDP-43 (Myc) was detected in the purified mitochondria 24 hr following induction of TDP-43 expression, and that the endogenous (Endo) TDP-43 was detected in the purified mitochondria from both control and TDP-43 expressing cells. Western Blotting experiments were performed as described for Fig 2A using total cell lysates (Total), cytoplasmic fractions (Cyto) or purified mitochondria (Mito) with the specific antibodies as indicated. (**B, C**). Quantification of mitochondrial size (B) or percentage of damaged mitochondria in EM analyses of the corresponding HEK293 cells, vector control (Ctr) or cells expressing Wt or A315T-mutant TDP-43. Approximately 200 mitochondria (Ctr: 200, Wt: 200, A315T: 201, respectively) were quantified for each group. Data represents 3 independent experiments [one-way ANOVA with Bonferroni post hoc test (***: *P*<0.001)].
(TIF)

S2 Fig **(A) Induction of either Wt or A315T-mutant TDP-43 expression does not affect the total cellular ATP level.** The total cellular ATP level was measured in cells expressing the control, or Wt or A315T-mutant TDP-43 at different time points after induction of TDP-43 expression using tetracycline (1ug/ml Tet). **(B) Induction of either Wt or A315T-mutant TDP-43 expression does not lead to a general reduction in the mRNA levels of respiratory complex I genes.** Quantitative RT-PCR experiment was performed at 24 hr post-induction using specific primers to examine the expression of a number of components of the complex I, including ND3, ND6, NDUFAB1, NDUFS8, NDUFAF4 and NDUFA13. Data represent 3 independent experiments and are analyzed using a one-way ANOVA with Bonferroni post hoc test (ns: not significant; *: *P*<0.05; **: *P*<0.01; ***: *P*<0.001).
(TIF)

S3 Fig Expression of A315T-mutant TDP-43 leads to progressive retinal neurodegeneration and mitochondrial damage in a heat-shock inducible fly model. TDP-43 expression was induced by heat shock in the retinae (GMR) or all neurons (Elav) under the Gal4 driver containing a Tubulin-Gal80ts regulatory element. **(A)** A diagram illustrating the heat-shock induction strategy. Adult flies were collected after eclosion and subjected to heat shock daily at 28°C for 4 hours followed by culturing at 25°C for 20 hours every day. **(B, C)** TEM analyses revealed that the expression of A315T-mutant TDP-43 under the GMR-Gal4/Tubulin-Gal80^ts^ driver leads to age-dependent progressive retinal degeneration and mitochondrial damage, whereas the retinae and mitochondria of control flies retained their normal morphology even at day 30 post-induction (Day30 post-Ind).
(TIF)

S4 Fig TDP-43 expression increases mitochondrial ROS levels in the motor neurons (MNs) of TDP-43 transgenic flies. **(A)** Confocal ratiometric imaging of VNC motor neurons of larvae expressing control RFP (Ctr), Wt or A315T-mutant TDP-43 revealed increased mitochondrial ROS production (excitation for reduced/oxidized mito-roGFP2-Grx1: 405-nm/488-nm). Scale bars: 25μm. **(B)** Quantification of the mitochondrial redox index (405-nm/488-nm) in MNs of fly larvae expressing Ctr, Wt or A315T-mutant TDP-43. Thirty-two to forty images were taken from more than 15 flies in each group (Ctr: 35 images from 18 flies; Wt: 40 from 20 flies; A315T: 32 from 16 flies, respectively). Data represent 3 independent experiments, analyzed using a one-way ANOVA with Bonferroni post hoc test (***:*P*<0.001). Fly genotypes: **Ctr**: OK371-Gal4/UAS-mito-roGFP2-Grx1/UAS-RFP; **Wt**: OK371-Gal4/UAS-mito-roGFP2-Grx1/UAS-Wt-TDP-43-RFP;
**A315T**: OK371-Gal4/UAS-mito-roGFP2-Grx1/UAS-A315T-TDP-43-RFP.
(TIF)

S5 Fig **(A, B) TDP-43-induced cytotoxicity is enhanced by the LonP1 inhibitor, but not by proteasome or autophagy inhibitors.** Control (Ctr) cells or cells expressing Wt or A315T-mutant TDP-43 were induced with Tet (1μg/mL) for 24 hours. Following PBS washes to remove Tet, cells were cultured for an additional 24hours in media containing LonP1 inhibitor CDDO (CD; 3μM), proteasome inhibitor MG132 (MG; 10μM) or autophagy inhibitor 3MA (MA; 3mM). Cell viability or cytotoxicity was determined using a CytoTox-ONE Homogeneous Membrane Integrity kit (Promega) in cells treated with CD, MG or MA. Data represent 4 independent experiments and are analyzed using StatPlus (one-way ANOVA with Bonferroni post hoc test). (**C) Increased LonP1 expression suppresses TDP-43 cytotoxicty (see Fig 7B) without altering the total TDP-43 levels.** Western blotting analyses show increased LonP1 expression following LonP1 transfection (+) in cells expressing control vector (Ctr), Wt or A315T-TDP-43. The total TDP-43 levels in cell lysates were not changed by LonP1 overexpression (OE). (**D) TDP-43 degradation by LonP1 is ATP-dependent.** The *in vitro* degradation assay was carried out as described for Fig 7E using purified recombinant LonP1 in the presence (+) or absence (-) of 5mM ATP and different concentrations of LonP1 protein (0, 0.5 or 1.5μM for “-, + or ++” respectively). In the absence of ATP, there was no detectable degradation of TDP-43 by LonP1. Data in panels C and D represent 3 independent experiments.
(TIF)

S6 Fig **(A) There was no detectable interaction between ClpP and TDP-43 in the co-Immunoprecipitation assay. (B) Down-regulating ClpP did not affect mitochondrial TDP-43 protein level.** ClpP was down-regulated in HEK293 stable inducible cells expressing Wt or A315T-mutant TDP-43. ClpP down-regulation did not alter mitochondrial TDP-43 levels. Data represent 3 independent experiments.
(TIF)

S7 Fig Total TDP-43 expression is unaffected by Lon knockdown in fly photoreceptors (GMR-Gal4) or in fly neurons (Elav-Gal4). (**A**) qPCR experiment was carried out to determine mRNA levels of Drosophila Lon (dLon) in the corresponding fly lines expressing vector control (Ctr) or siLon (#1 and #2) or overexpressing Lon (dLonOE). Because siLon#1 line showed consistently more robust down-regulation (reducing dLon expression to ~30% of the control level), this line was used in subsequent experiments. Data represent 3 independent experiments. Overexpression of dLon in control flies led to retinal degeneration, preventing us from testing effect of dLonOE in TDP-43 flies. (**B**) Western blotting experiments using anti-TDP-43 antibody showed that the total TDP-43 was expressed in eyes at equivalent levels in TDP-43 and TDP-43/siLon flies at day 20 following heat shock. (**C**) Western blotting experiments using anti-TDP-43 antibody showed that the total TDP-43 was expressed at equivalent levels in heads of TDP-43 and TDP-43/siLon flies at day 4 following heat shock. The pan-neuronal marker Elav was used as a loading control in panels B and C.
(TIF)

S8 Fig Down-regulating Lon increased mitochondrial TDP-43 level, including detergent-insoluble fractions. **(A).** Mitochondria were purified from heads of flies expressing control or Wt or A315T-mutant TDP-43 under the GMR-Gal4 driver. Purified mitochondria were sequentially extracted in RIPA buffers containing 0.5% NP-40 or 2%SDS and finally 8M Urea (see Materials and Methods). Corresponding NP-40 soluble, or NP-40 resistant/SDS-soluble, or SDS-resistant/Urea-soluble fractions (lanes 1–4, 5–8 or 9–12 respectively) were analyzed by Western blotting using anti-TDP-43 and anti-ATP5A1. (**B**). Quantification of WB band intensity shown in panel A. Data from 3 experiments were analyzed by a Student’s *t*-test. Data represent 3 independent experiments.
(TIF)

S9 Fig Quantification of EM data shows that down-regulation of Lon in control flies does not affect rhabdomere formation or mitochondrial morphology. **(A)** Down-regulation of Lon in control (Ctr) flies did not affect retinal morphology or mitochondrial ultra-structure. **(B)** Down-regulating Lon in control flies did not affect the number of rhabdomeres per ommatidium, with 81 and 89 ommatidia analyzed in Ctr and siLon groups respectively. **(C)** Quantification of EM data shows that down-regulating Lon in control flies did not alter the percentage of damaged mitochondria, with 431 mitochondria in Ctr group and 521 mitochondria in siLon group examined. Data represent 3 independent experiments, analyzed using a one-way ANOVA with Bonferroni post hoc test (*: *P*<0.05; **: *P*<0.01).
(TIF)

S10 Fig Electron-dense aggregates were detected inside mitochondria in flies expressing A315T-mutant TDP-43 A315T when Lon was down-regulated. Electron-dense aggregates (marked by black arrows) were detected inside mitochondria in flies expressing A315T-mutant TDP-43 when Lon was down-regulated by the specific siRNA. These structures were not detected in other groups, including the control flies or flies expressing Wt (with or without Lon knockdown) or A315T-mutant TDP-43 alone.
(TIF)
