# Supplementary material for: TDP-43 induces mitochondrial damage and activates the mitochondrial unfolded protein response
Source: PLoS Genet. 2019 May 17;15(5):e1007947. doi: 10.1371/journal.pgen.1007947 (PMC6524796; doi:10.1371/journal.pgen.1007947)
Supplement: S1 Table — All samples used were sequenced and confirmed that there were no mutations in known genes associated with ALS or FTLD, including TDP-43, FUS, C9orf72, GRN, SOD1 and MAPT, as reported previously [40]. Age, gender, Post-mortem interval (PMI; hours), together with pathological and clinical diagnoses, are included. (DOCX) [file pgen.1007947.s001.docx]

**Supplementary Table S1**

**Case # Age Sex PMI Tissue (Pathological Diagnosis) Clinical Diagnosis**

Ctr#1 72 M 14 Temporal (no TDP-43 pathology) Non-demented, Thyroid cancer

Ctrl#2 77 M 17 Temporal (no TDP-43 pathology) Pulmonary fibrosis

Ctrl#3 57 F 14 Temporal (no TDP-43 pathology) Non-demented

FTLD#1 65 F 5 Temporal lobe (FTLD-TDP type C) FTD

FTLD#2: 43 F 7 Hippocampus (ALS + FTLD-TDP) ALS+FTD

FTLD#3 59 F 21 Temporal lobe (FTLD-TDP type B + ALS) FTD+ALS

FTLD#4: 59 M 12 Temporal lobe (FTLD-TDP type B + ALS) PPA

FTLD#5 58 M 16 Dentate gyrus (ALS + FTLD-TDP type B) ALS+dementia
