# Supplementary material for: TDP-43 induces mitochondrial damage and activates the mitochondrial unfolded protein response
Source: PLoS Genet. 2019 May 17;15(5):e1007947. doi: 10.1371/journal.pgen.1007947 (PMC6524796; doi:10.1371/journal.pgen.1007947)
Supplement: S2 Table — (DOCX) [file pgen.1007947.s002.docx]

**Supplemental Table S2. Primers used in qPCR experiments.**

| Gene | Direction | Primer sequence | Species |
| --- | --- | --- | --- |
| ATF5 | forward | 5’-CTGGCTCCCTATGAGGTCCTTG-3 | *H. sapiens* |
|  | reverse | 5’-GAGCTGTGAAATCAACTCGCTCAG-3’ |  |
| HSP60 | forward | 5’-GATGCTGTGGCCGTTACAATG-3’ |  |
|  | reverse | 5’-GTCAATTGACTTTGCAACAGTCACAC-3’ |  |
| HSPA9 | forward | 5’-CAAGCGACAGGCTGTCACCAAC-3’ |  |
|  | reverse | 5’-CAACCCAGGCATCACCATTGG-3’ |  |
| LonP1 | forward | 5’-CATTGCCTTGAACCCTCTC-3’ |  |
|  | reverse | 5’-ATGTCGCTCAGGTAGATGG-3’ |  |
| HPRT | forward | 5’-CTTTGCTGACCTGCTGGATT-3’ |  |
|  | reverse | 5’-TCCCCTGTTGACTGGTCATT-3’ |  |
| ND3 | forward | 5’-CAAACAACTAACCTGCCACT-3’ |  |
|  | reverse | 5’-TTCGGTTCAGTCTAATCCTT-3’ |  |
| ND6 | forward | 5’-GTGCTGTGGGTGAAAGAG-3’ |  |
|  | reverse | 5’-CGCCCATAATCATACAAA-3’ |  |
| NDUFAB1 | forward | 5’-TGCAGATAAGAAGGATGT-3’ |  |
|  | reverse | 5’-TCAGAGTCAGCAAGAATG-3’ |  |
| NDUFAF4 | forward | 5’-TGATTCCAAAGATCCTGT-3’ |  |
|  | reverse | 5’-AAGTGTCAATGCTTCTAC-3’ |  |
| NDUFS8 | forward | 5’-GCATCTACTGCGGCTTCTG-3’ |  |
|  | reverse | 5’-GTAGTCAGCCTGGATGTTGG-3’ |  |
| NDUFA13 | forward | 5'-ACCTTGCAGATGCTTCGGGAGA-3' |  |
|  | reverse | 5'-AAGCCGTGGCTGGCATGGAG-3' |  |
| HSP60 | forward | 5'–CACAGAAAAGTCAAGCGAACTG–3' | *D. melanogaster* |
|  | reverse | 5'–GAAACTGGCAAACGGAACATC–3' |  |
| Hsc70-5 | forward | 5'–AAGTGTCGCTCGAACTGC–3' |  |
|  | reverse | 5'–GAGGTCAGGAAAGCCACTTC–3' |  |
| CG5045 | forward | 5'–GATCATGCTGAAAACCGCTG–3' |  |
|  | reverse | 5'–CGTGAGAATATGTCGTAGGCC–3' |  |
| Lon-RA | forward | 5'-CCAGTCTCAGGTTCCACTATC-3' |  |
|  | reverse | 5'-CTAAGCCCGCTGAAGATCAAA-3' |  |
| Lon-RC | forward | 5'-TGACAACTTTGCATTATCCTCT-3' |  |
|  | reverse | 5'-GACTCGACTTTGCCTGATTT-3' |  |
| Actin5C | forward | 5'-GCGTTTTGTACAATTCGTCAGCAACC-3' |  |
|  | reverse | 5'-GCACGCGAAACTGCAGCCAA-3' |  |
