# Supplementary figures and images for: TDP-43 induces mitochondrial damage and activates the mitochondrial unfolded protein response
Source: PLoS Genet. 2019 May 17;15(5):e1007947. doi: 10.1371/journal.pgen.1007947 (PMC6524796; doi:10.1371/journal.pgen.1007947)

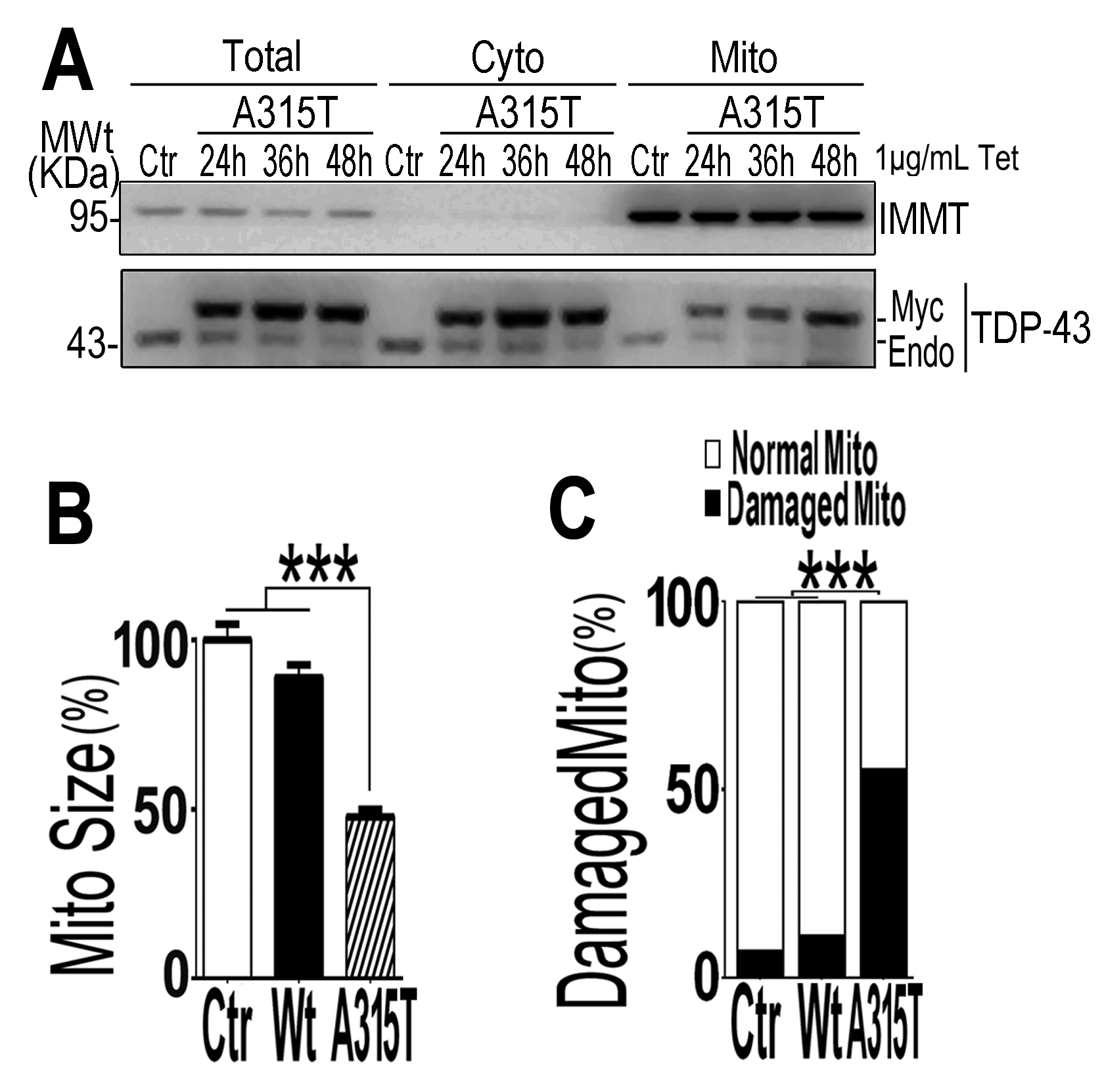

Supplement: S1 Fig — (A) Western blotting experiments revealed that the Myc-tagged TDP-43 (Myc) was detected in the purified mitochondria 24 hr following induction of TDP-43 expression, and that the endogenous (Endo) TDP-43 was detected in the purified mitochondria from both control and TDP-43 expressing cells. Western Blotting experiments were performed as described for Fig 2A using total cell lysates (Total), cytoplasmic fractions (Cyto) or purified mitochondria (Mito) with the specific antibodies as indicated. (B, C). Quantification of mitochondrial size (B) or percentage of damaged mitochondria in EM analyses of the corresponding HEK293 cells, vector control (Ctr) or cells expressing Wt or A315T-mutant TDP-43. Approximately 200 mitochondria (Ctr: 200, Wt: 200, A315T: 201, respectively) were quantified for each group. Data represents 3 independent experiments [one-way ANOVA with Bonferroni post hoc test (***: P<0.001)]. (TIF) [file pgen.1007947.s003.tif]

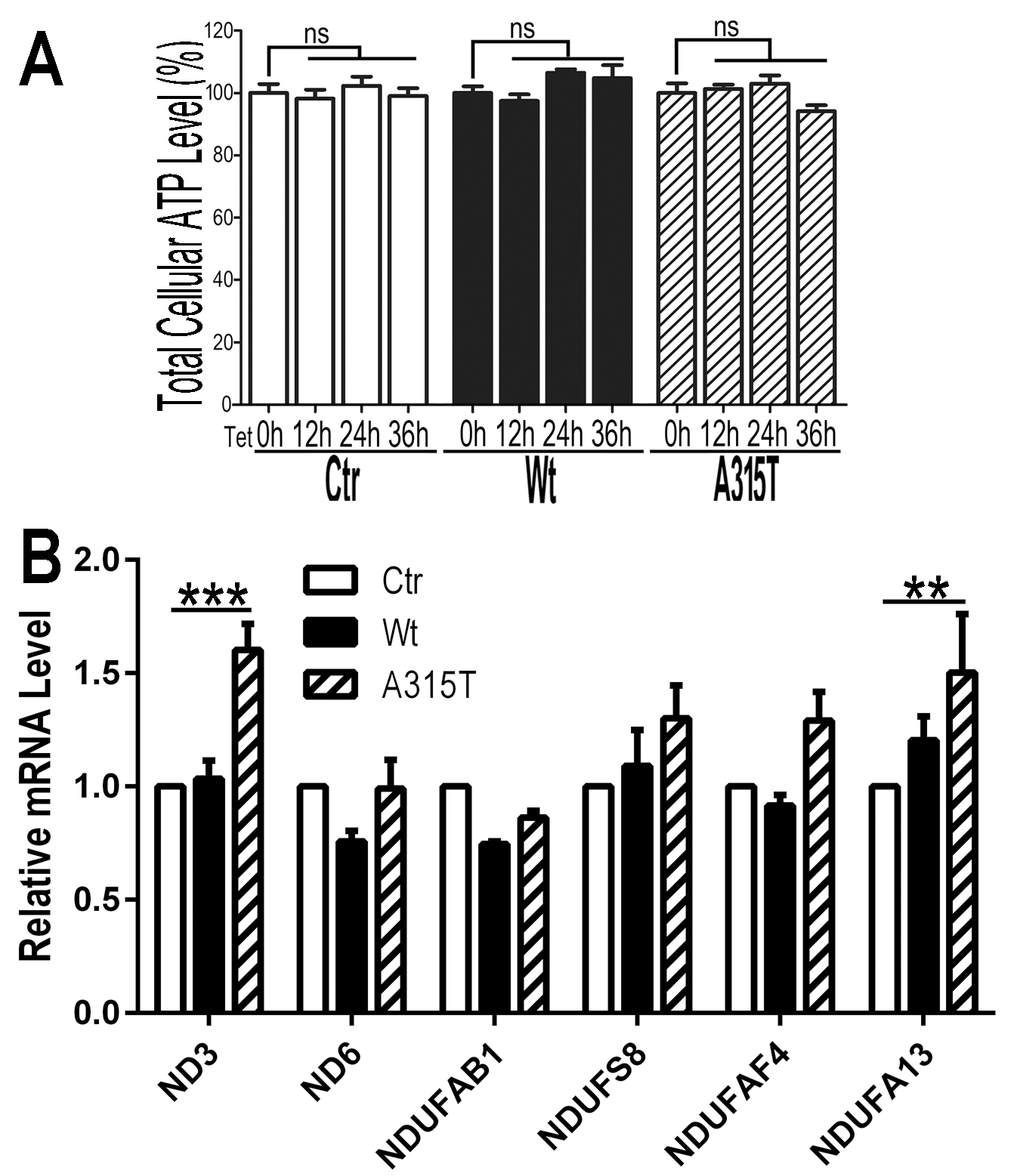

Supplement: S2 Fig — (A) Induction of either Wt or A315T-mutant TDP-43 expression does not affect the total cellular ATP level. The total cellular ATP level was measured in cells expressing the control, or Wt or A315T-mutant TDP-43 at different time points after induction of TDP-43 expression using tetracycline (1ug/ml Tet). (B) Induction of either Wt or A315T-mutant TDP-43 expression does not lead to a general reduction in the mRNA levels of respiratory complex I genes. Quantitative RT-PCR experiment was performed at 24 hr post-induction using specific primers to examine the expression of a number of components of the complex I, including ND3, ND6, NDUFAB1, NDUFS8, NDUFAF4 and NDUFA13. Data represent 3 independent experiments and are analyzed using a one-way ANOVA with Bonferroni post hoc test (ns: not significant; *: P<0.05; **: P<0.01; ***: P<0.001). (TIF) [file pgen.1007947.s004.tif]

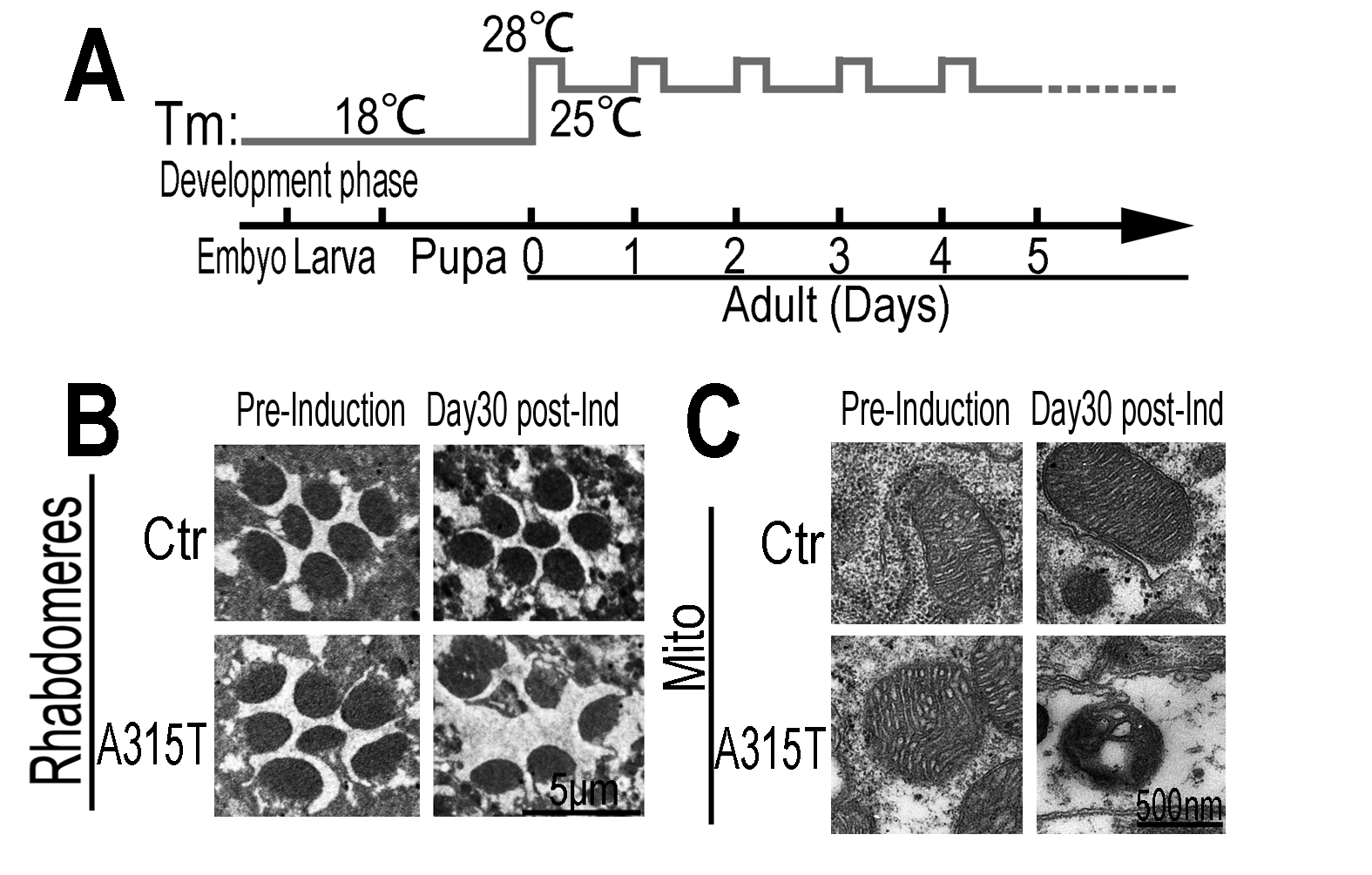

Supplement: S3 Fig — TDP-43 expression was induced by heat shock in the retinae (GMR) or all neurons (Elav) under the Gal4 driver containing a Tubulin-Gal80ts regulatory element. (A) A diagram illustrating the heat-shock induction strategy. Adult flies were collected after eclosion and subjected to heat shock daily at 28°C for 4 hours followed by culturing at 25°C for 20 hours every day. (B, C) TEM analyses revealed that the expression of A315T-mutant TDP-43 under the GMR-Gal4/Tubulin-Gal80ts driver leads to age-dependent progressive retinal degeneration and mitochondrial damage, whereas the retinae and mitochondria of control flies retained their normal morphology even at day 30 post-induction (Day30 post-Ind). (TIF) [file pgen.1007947.s005.tif]

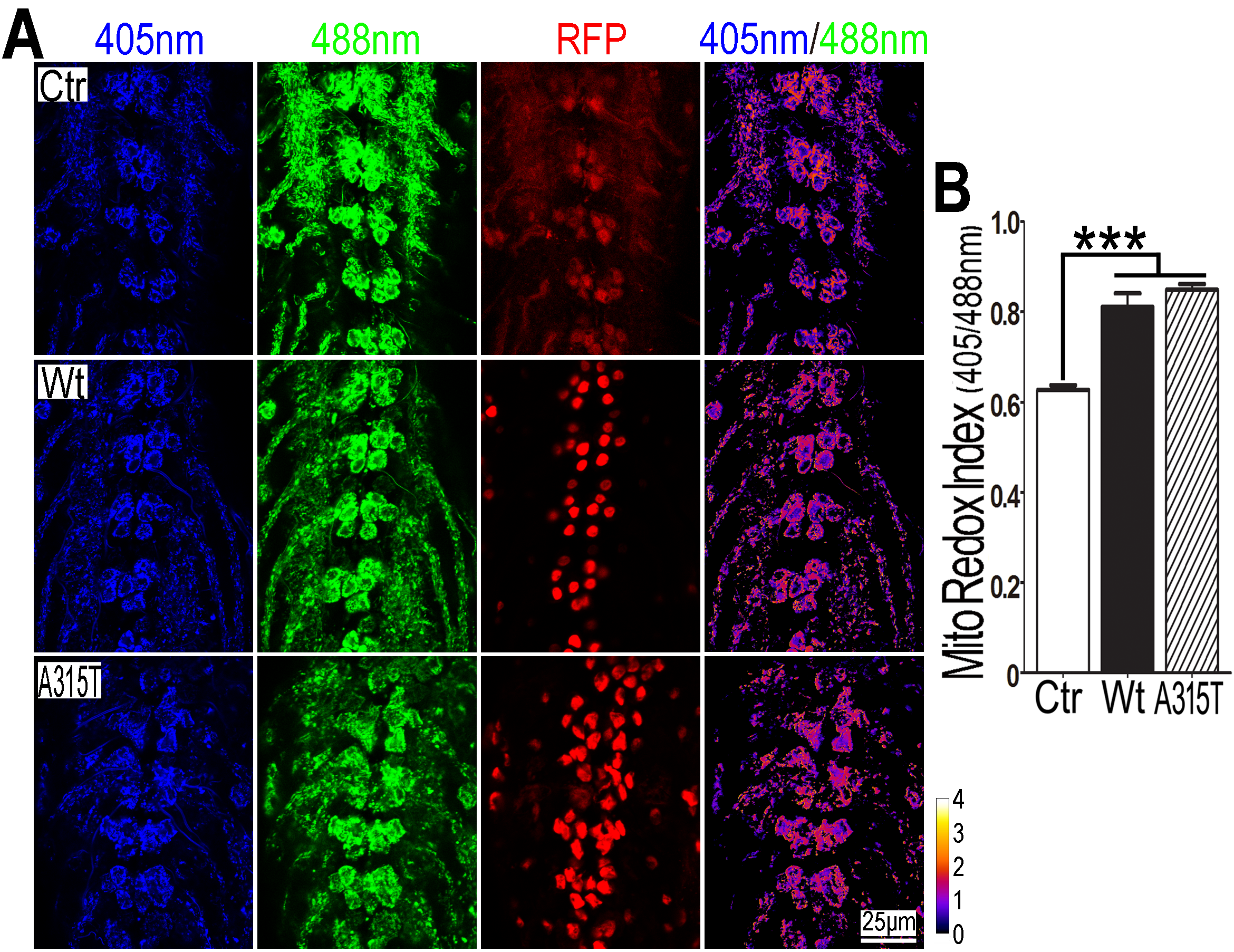

Supplement: S4 Fig — (A) Confocal ratiometric imaging of VNC motor neurons of larvae expressing control RFP (Ctr), Wt or A315T-mutant TDP-43 revealed increased mitochondrial ROS production (excitation for reduced/oxidized mito-roGFP2-Grx1: 405-nm/488-nm). Scale bars: 25μm. (B) Quantification of the mitochondrial redox index (405-nm/488-nm) in MNs of fly larvae expressing Ctr, Wt or A315T-mutant TDP-43. Thirty-two to forty images were taken from more than 15 flies in each group (Ctr: 35 images from 18 flies; Wt: 40 from 20 flies; A315T: 32 from 16 flies, respectively). Data represent 3 independent experiments, analyzed using a one-way ANOVA with Bonferroni post hoc test (***:P<0.001). Fly genotypes: Ctr: OK371-Gal4/UAS-mito-roGFP2-Grx1/UAS-RFP; Wt: OK371-Gal4/UAS-mito-roGFP2-Grx1/UAS-Wt-TDP-43-RFP; A315T: OK371-Gal4/UAS-mito-roGFP2-Grx1/UAS-A315T-TDP-43-RFP. (TIF) [file pgen.1007947.s006.tif]

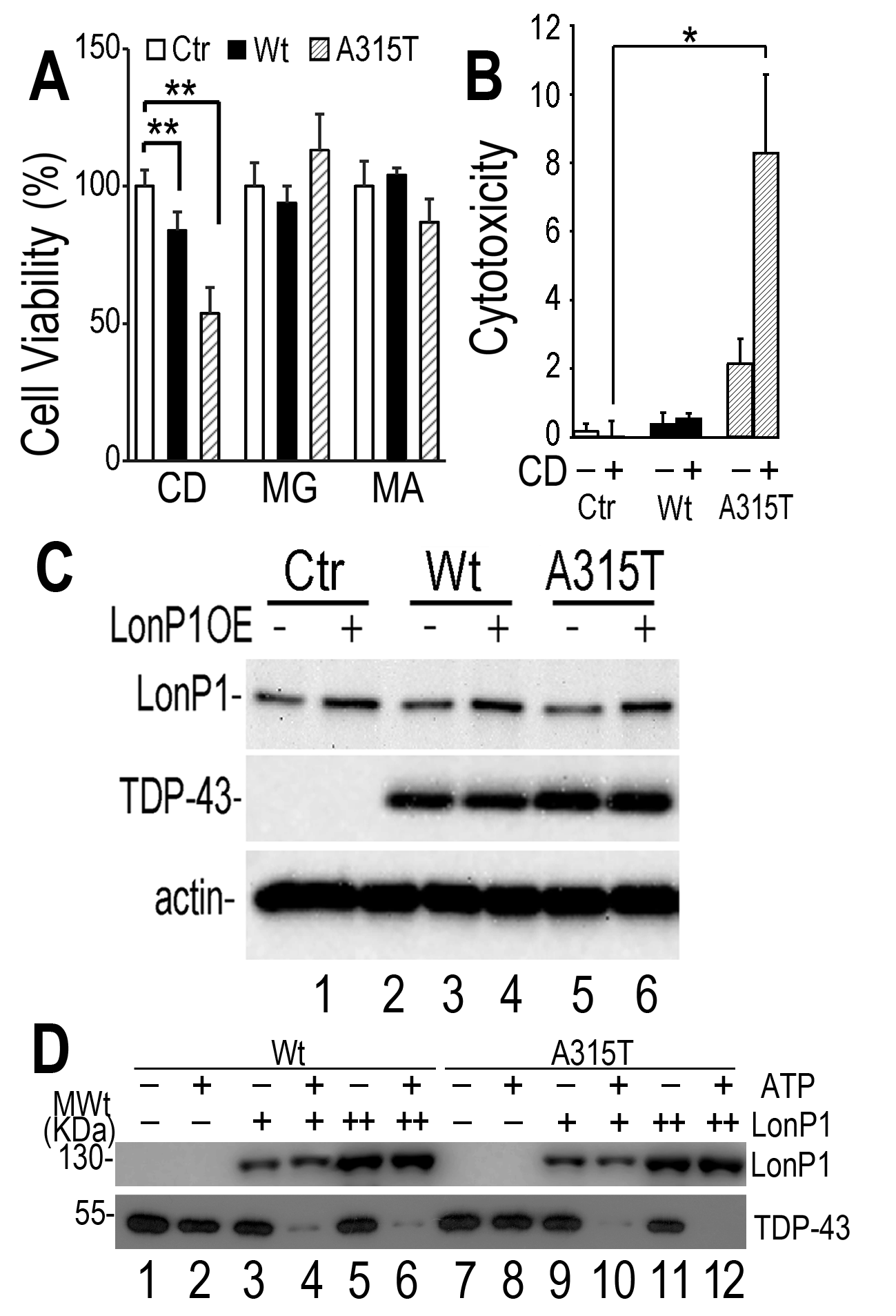

Supplement: S5 Fig — (A, B) TDP-43-induced cytotoxicity is enhanced by the LonP1 inhibitor, but not by proteasome or autophagy inhibitors. Control (Ctr) cells or cells expressing Wt or A315T-mutant TDP-43 were induced with Tet (1μg/mL) for 24 hours. Following PBS washes to remove Tet, cells were cultured for an additional 24hours in media containing LonP1 inhibitor CDDO (CD; 3μM), proteasome inhibitor MG132 (MG; 10μM) or autophagy inhibitor 3MA (MA; 3mM). Cell viability or cytotoxicity was determined using a CytoTox-ONE Homogeneous Membrane Integrity kit (Promega) in cells treated with CD, MG or MA. Data represent 4 independent experiments and are analyzed using StatPlus (one-way ANOVA with Bonferroni post hoc test). (C) Increased LonP1 expression suppresses TDP-43 cytotoxicty (see Fig 7B) without altering the total TDP-43 levels. Western blotting analyses show increased LonP1 expression following LonP1 transfection (+) in cells expressing control vector (Ctr), Wt or A315T-TDP-43. The total TDP-43 levels in cell lysates were not changed by LonP1 overexpression (OE). (D) TDP-43 degradation by LonP1 is ATP-dependent. The in vitro degradation assay was carried out as described for Fig 7E using purified recombinant LonP1 in the presence (+) or absence (-) of 5mM ATP and different concentrations of LonP1 protein (0, 0.5 or 1.5μM for “-, + or ++” respectively). In the absence of ATP, there was no detectable degradation of TDP-43 by LonP1. Data in panels C and D represent 3 independent experiments. (TIF) [file pgen.1007947.s007.tif]

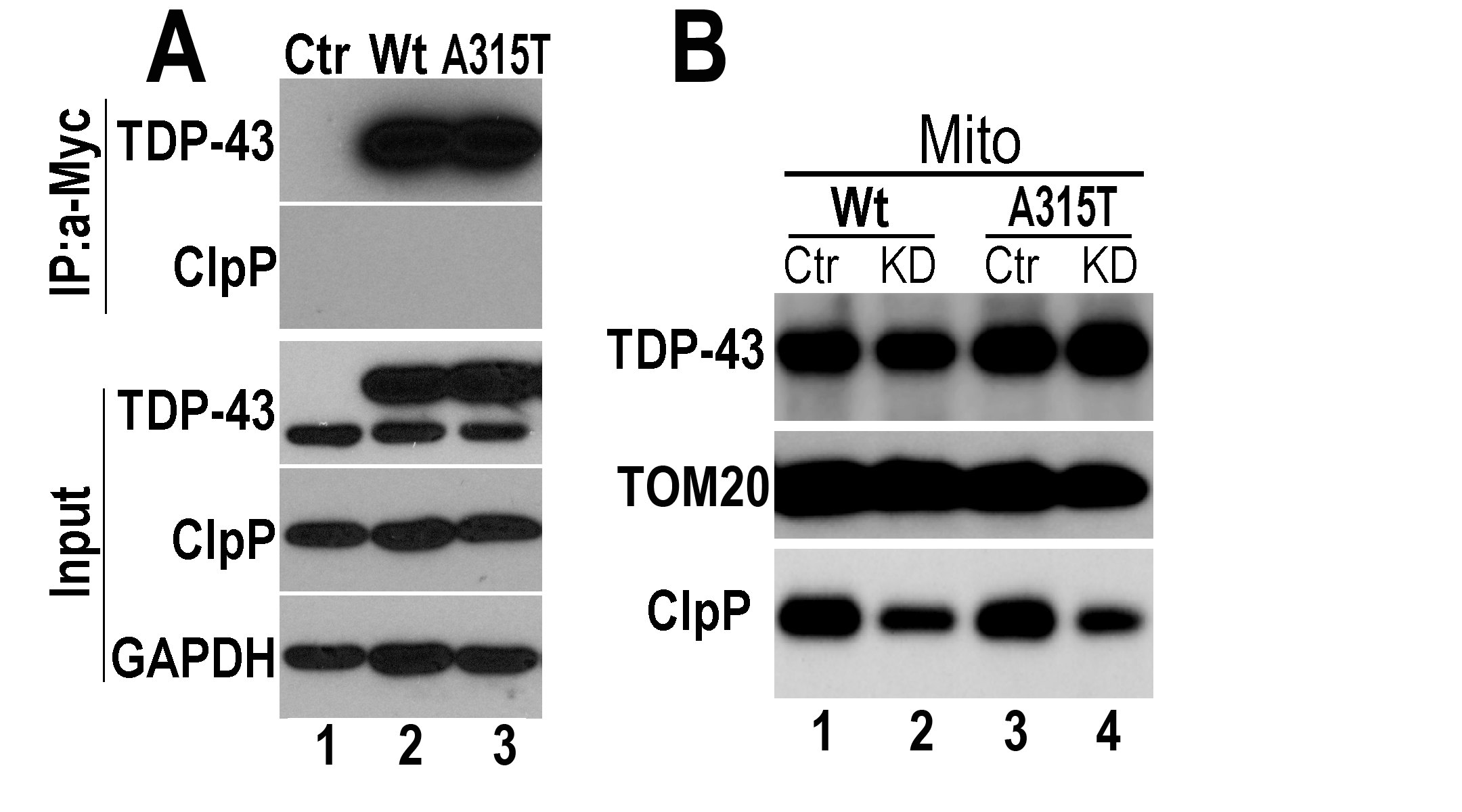

Supplement: S6 Fig — (A) There was no detectable interaction between ClpP and TDP-43 in the co-Immunoprecipitation assay. (B) Down-regulating ClpP did not affect mitochondrial TDP-43 protein level. ClpP was down-regulated in HEK293 stable inducible cells expressing Wt or A315T-mutant TDP-43. ClpP down-regulation did not alter mitochondrial TDP-43 levels. Data represent 3 independent experiments. (TIF) [file pgen.1007947.s008.tif]

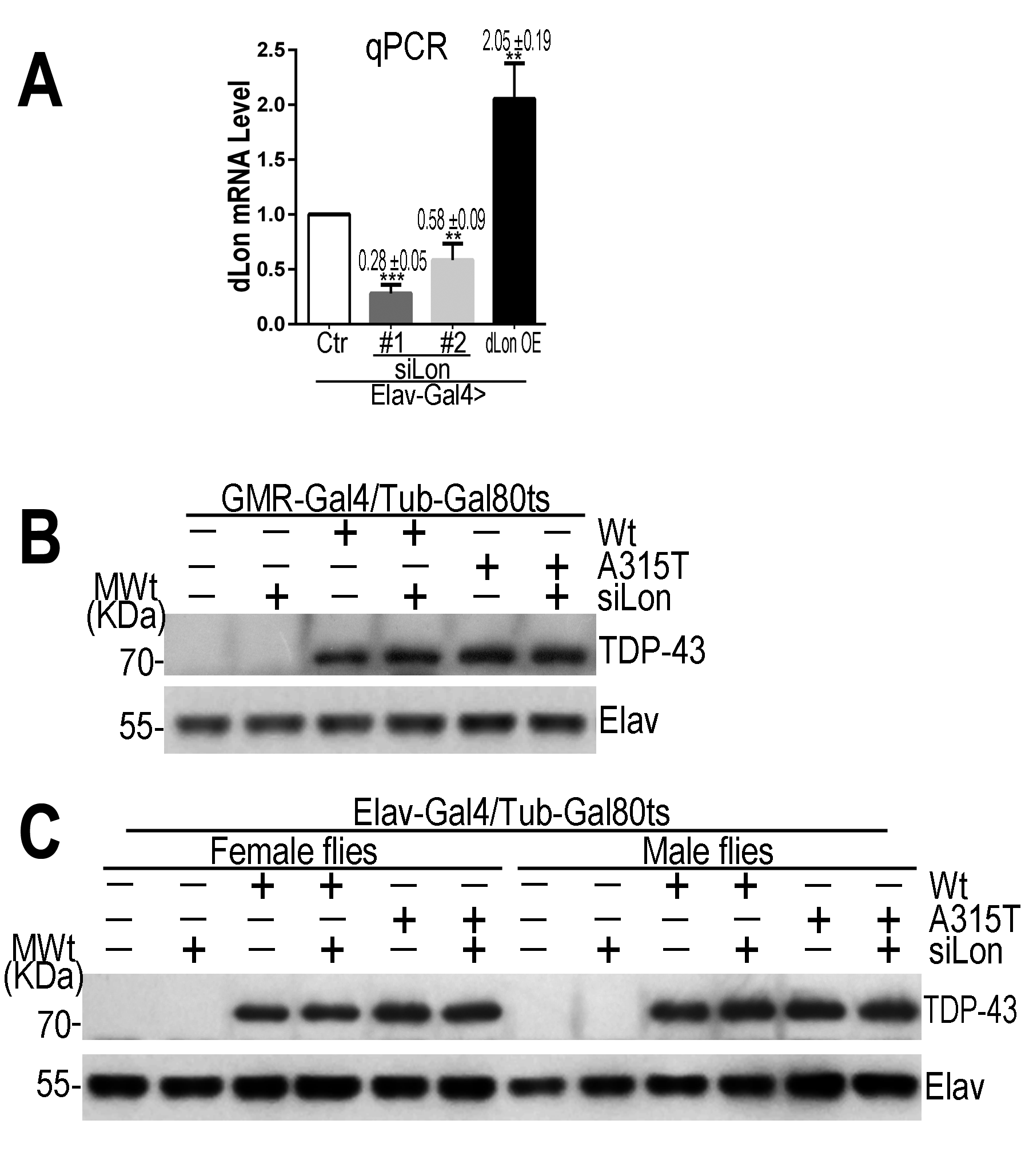

Supplement: S7 Fig — (A) qPCR experiment was carried out to determine mRNA levels of Drosophila Lon (dLon) in the corresponding fly lines expressing vector control (Ctr) or siLon (#1 and #2) or overexpressing Lon (dLonOE). Because siLon#1 line showed consistently more robust down-regulation (reducing dLon expression to ~30% of the control level), this line was used in subsequent experiments. Data represent 3 independent experiments. Overexpression of dLon in control flies led to retinal degeneration, preventing us from testing effect of dLonOE in TDP-43 flies. (B) Western blotting experiments using anti-TDP-43 antibody showed that the total TDP-43 was expressed in eyes at equivalent levels in TDP-43 and TDP-43/siLon flies at day 20 following heat shock. (C) Western blotting experiments using anti-TDP-43 antibody showed that the total TDP-43 was expressed at equivalent levels in heads of TDP-43 and TDP-43/siLon flies at day 4 following heat shock. The pan-neuronal marker Elav was used as a loading control in panels B and C. (TIF) [file pgen.1007947.s009.tif]

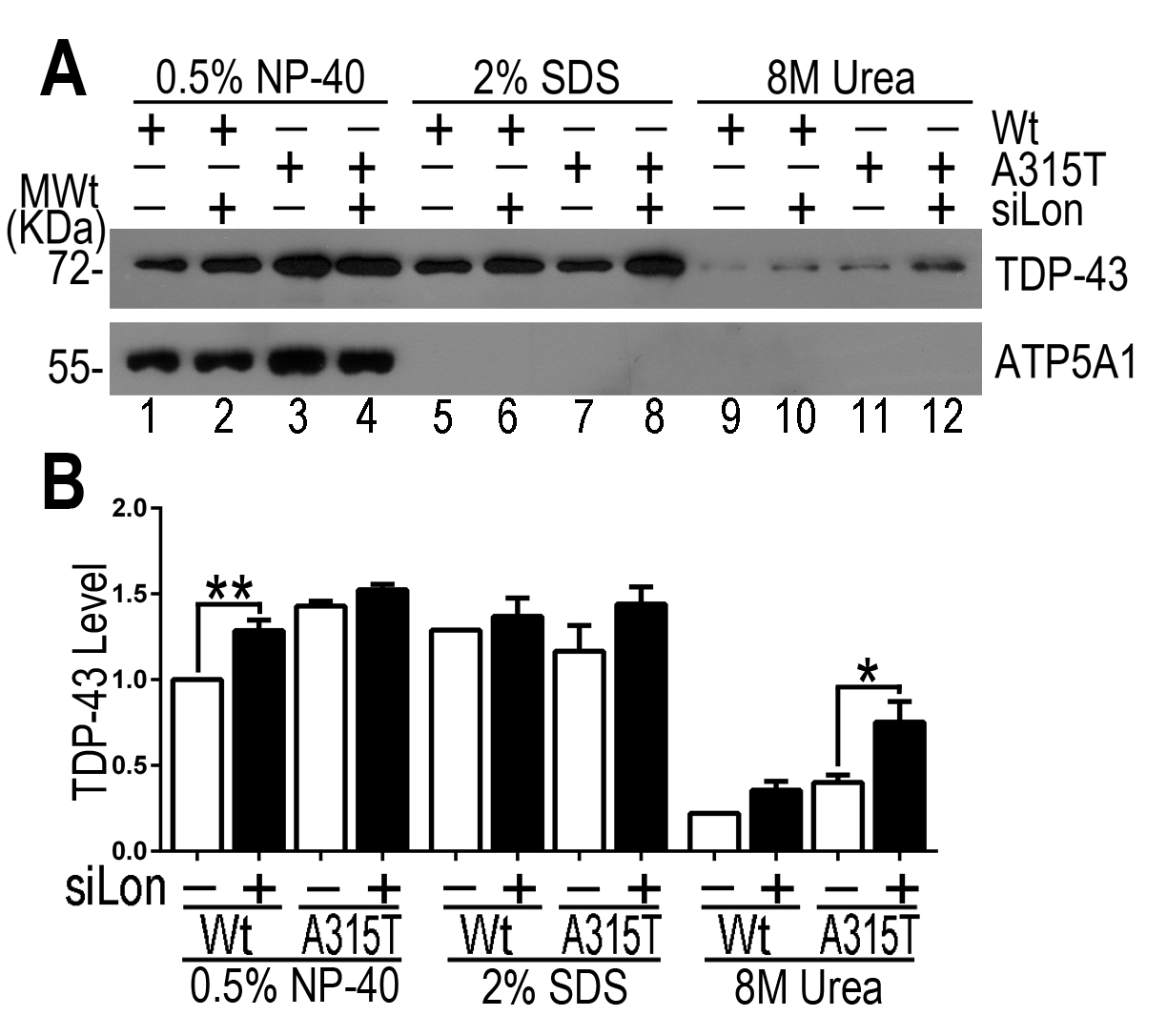

Supplement: S8 Fig — (A). Mitochondria were purified from heads of flies expressing control or Wt or A315T-mutant TDP-43 under the GMR-Gal4 driver. Purified mitochondria were sequentially extracted in RIPA buffers containing 0.5% NP-40 or 2%SDS and finally 8M Urea (see Materials and Methods). Corresponding NP-40 soluble, or NP-40 resistant/SDS-soluble, or SDS-resistant/Urea-soluble fractions (lanes 1–4, 5–8 or 9–12 respectively) were analyzed by Western blotting using anti-TDP-43 and anti-ATP5A1. (B). Quantification of WB band intensity shown in panel A. Data from 3 experiments were analyzed by a Student’s t-test. Data represent 3 independent experiments. (TIF) [file pgen.1007947.s010.tif]

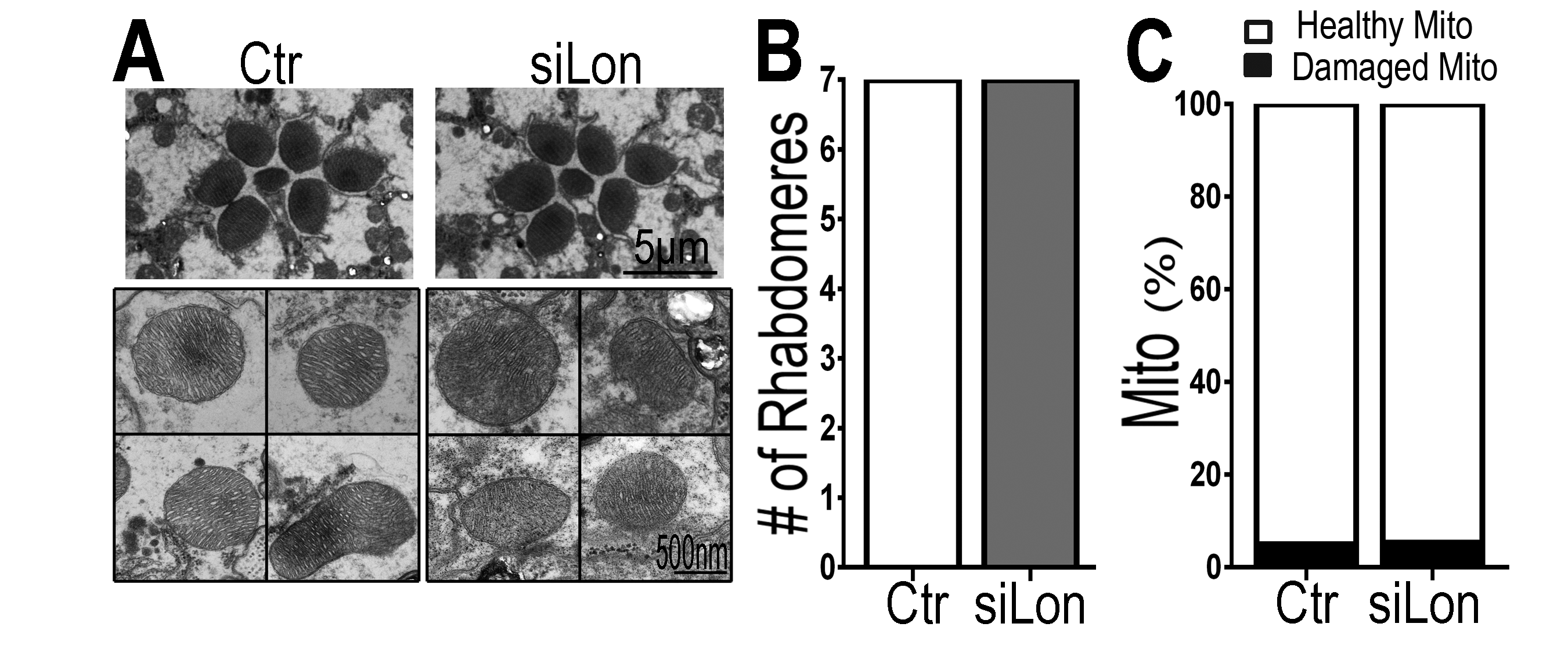

Supplement: S9 Fig — (A) Down-regulation of Lon in control (Ctr) flies did not affect retinal morphology or mitochondrial ultra-structure. (B) Down-regulating Lon in control flies did not affect the number of rhabdomeres per ommatidium, with 81 and 89 ommatidia analyzed in Ctr and siLon groups respectively. (C) Quantification of EM data shows that down-regulating Lon in control flies did not alter the percentage of damaged mitochondria, with 431 mitochondria in Ctr group and 521 mitochondria in siLon group examined. Data represent 3 independent experiments, analyzed using a one-way ANOVA with Bonferroni post hoc test (*: P<0.05; **: P<0.01). (TIF) [file pgen.1007947.s011.tif]

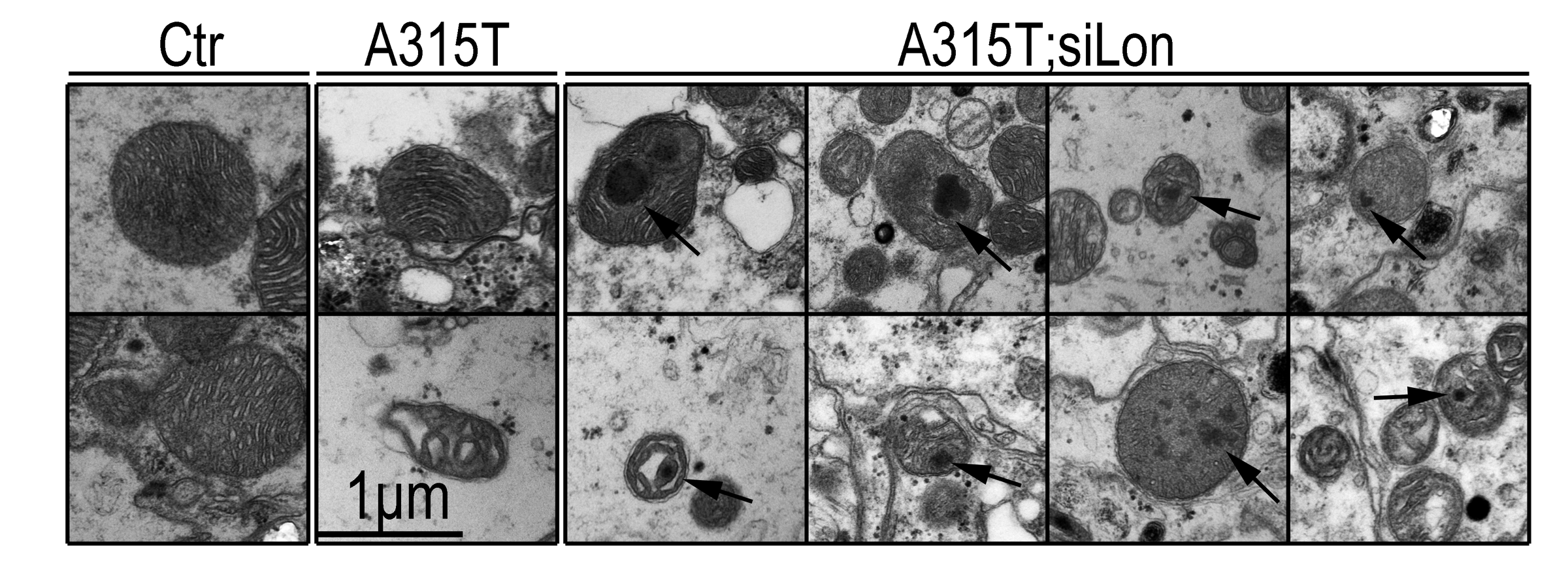

Supplement: S10 Fig — Electron-dense aggregates (marked by black arrows) were detected inside mitochondria in flies expressing A315T-mutant TDP-43 when Lon was down-regulated by the specific siRNA. These structures were not detected in other groups, including the control flies or flies expressing Wt (with or without Lon knockdown) or A315T-mutant TDP-43 alone. (TIF) [file pgen.1007947.s012.tif]
